# Oxalate induces proliferation and mitochondrial metabolism in select clear cell renal cell carcinoma cell lines

**DOI:** 10.1186/s12885-026-15847-0

**Published:** 2026-03-17

**Authors:** Garrett Hendley, Parveen Kumar, Vivek Verma, Mary Doamekpor, Natalie R. Gassman, Christine M. Wright, Hyeyoung Nam, Chen-Han Wilfred Wu, Tanecia Mitchell

**Affiliations:** 1 Department of Urology, University of Alabama Birmingham, Hugh Kaul Human Genetics Building, 840B, 720 20th Street South, Birmingham, AL 35294 USA; 2 Department of Pathology, University of Alabama Birmingham, Birmingham, AL USA

**Keywords:** Oxalate, Clear Cell Renal Cell Carcinoma, Metabolism, DNA damage, Reactive Oxygen Species

## Abstract

Renal Cell Carcinoma (RCC) leads to over $3 billion in annual healthcare costs and reduces patients' quality of life. While the causes of RCC are not fully understood, studies suggest a link to kidney stone (KS) disease, particularly those containing calcium oxalate (CaOx). Oxalate is found in certain plant-derived foods and is known to cause oxidative stress, inflammation, and cancer-like properties in renal epithelial cells. However, its effect on RCC remains unclear. This study investigated the impact of oxalate on clear cell RCC (ccRCC), the most common RCC subtype, using two ccRCC cell lines: 786-O (ccB subtype) and 769-P (ccA subtype). We hypothesized that oxalate promotes tumor-like behavior in ccRCC cells. After exposing cells to both soluble and insoluble oxalate for 24 h, we measured cell proliferation, metabolism, oxidative stress, and DNA damage. In 786-O cells, oxalate increased proliferation, mitochondrial gene expression, and metabolism, while reducing oxidative stress. In contrast, oxalate had little effect on 769-P proliferation or metabolism but increased oxidative stress and DNA damage. To determine if these effects were subtype-specific, we treated Caki-1 cells (another ccB subtype) and observed similar responses to 786-O cells. Importantly, rapamycin, an mTOR inhibitor, prevented oxalate-induced responses in 786-O and Caki-1 cells. These findings suggest that oxalate enhances proliferation and metabolic activity in certain ccRCC subtypes and may contribute to the association between RCC and KS disease.

**Supplementary Information:**

The online version contains supplementary material available at 10.1186/s12885-026-15847-0.

## Background

Renal cell carcinoma (RCC) is a common cancer that results in significant annual healthcare costs (> $3 billion/year) [1]. In 2023, the United States was estimated to have 81,800 new RCC cases and 14,890 deaths from RCC [2]. Clear cell RCC (ccRCC) is the most common histological subtype of renal cancer and accounts for approximately 70–75% of cases [3]. ccRCC is localized to epithelial cells surrounding the renal tubules and can metastasize throughout the body, subsequently causing additional complications and reduced quality of life for patients. Specifically, patients with ccRCC can experience sharp pains in their abdomen, blood in their urine, persistent fever, fatigue, renal failure, and death [4, 5]. Unfortunately, the mechanisms contributing to the progression of ccRCC remain undefined. However, several factors including genetics, inflammation, the tumor microenvironment, metabolism, and diet, are all suggested to stimulate tumor growth, metastasis, and resistance to treatment [6, 7].

Oxalate is a small molecule found in certain vegetables, nuts, fruits, and teas [8]. It exists in both the soluble and insoluble crystalline forms and can be absorbed in the intestine and excreted in the urine [9–11]. In addition, oxalate can be produced endogenously. It is well established that continuous consumption of oxalate rich foods is a significant risk factor for the development of kidney stones (KS), particularly those comprised of calcium oxalate (CaOx) crystals [10]. We and others have determined that oxalate causes oxidative stress and inflammation in renal epithelial cells [12–15] as well as impairs mitochondrial function in immune cells [16–19].

Intact mitochondrial function is critical for cellular proliferation, metabolism, cell death, calcium homeostasis, redox homeostasis, cell signaling, and many other cellular processes [20]. Mitochondrial dysfunction frequently occurs in cancer cells through the “Warburg Effect”, which causes cancer cells to meet metabolic ATP demands primarily through glycolysis [20]. This is in contrast to non-carcinogenic cells that rely on mitochondrial respiration to produce ATP. Further, cancer cells have metabolic plasticity, which allows them to adapt to various energy demands. Cancer cells can also exhibit elevated mitochondrial reactive oxygen species (ROS) levels, which promote cellular processes such as proliferation, epithelial-mesenchymal transition, and cell survival [21]. However, excessive accumulation of ROS within cells can result in oxidative stress and cellular damage, including DNA mutations [3].

While a potential link between RCC and KS [22–27] exists, the cellular mechanisms driving this association remain to be fully elucidated. A potential factor involved in this association is oxalate. CaOx crystals have been observed in renal tumors and the stromas of patients [26]. A recent report described that CaOx crystals induce epithelial-mesenchymal transition and mesenchymal marker expression as well as decrease the expression of epithelial markers in non-cancerous renal cells [27]. In another study, oxalate was shown to be elevated in human breast tumor tissues compared to non-cancerous breast tissue and to induce breast cancer proliferation in mice and cell lines [28]. Further, the authors stated that free oxalate and not CaOx crystals, cause varied responses in two different breast cancer cell lines [28].

Based on the current literature, we chose to investigate the role of oxalate in ccRCC using two well-established human ccRCC cell lines, 786-O and 769-P. These cell lines are widely used in ccRCC research due to their consistent genotypic and phenotypic characteristics that recapitulate key features of the disease. Notably, both 786-O and 769-P harbor mutations in the von Hippel-Lindau (*VHL*) gene, a hallmark genetic alteration in ccRCC [29]. In addition, these cell lines have distinct molecular and phenotypic differences. For example, 786-O cells display a clear cell type B (ccB) phenotype; whereas, 769-P cells display a clear cell type A (ccA) phenotype [30]. ccA and ccB are two distinct molecular subtypes of ccRCC in humans and have been proposed as useful biomarkers for predicting patient prognosis [31]. Specifically, individuals with a ccB phenotype have poorer outcomes [32]. Lastly, 786-O cells can form spheres in non-adherent conditions compared to 769-P cell lines, which suggests 786-O cells have the ability to self-renew and have stem-like or tumor initiating properties [33].

Given the emerging evidence linking oxalate to metabolic dysregulation, oxidative stress, and even cancer-like properties, we postulated that oxalate could influence ccRCC. By using these cell lines, we aimed to gain mechanistic insights into whether soluble oxalate (NaOx) and insoluble CaOx crystals could modulate ccRCC cell proliferation, molecular signaling, and metabolism. Our findings show that oxalate induces proliferation and metabolism in 786-O cells and does not elicit similar responses in 769-P cells. We further validated that oxalate also induced similar findings in Caki-1 cells, another ccB subtype of ccRCC cell line. Lastly, we investigated the potential role of mTOR signaling in this process as the pathway is important for several cellular processes, such as proliferation and metabolism, and is frequently overactive in cancer. We determined that the inhibition of the mTOR signaling pathway prevented oxalate-mediated responses in both 786-O and Caki-1 cell lines. These findings suggest that oxalate promotes proliferation and metabolism in certain ccRCC subtypes and may contribute to the association between RCC and KS disease.

## Methods

### Reagents and materials

Reagents were purchased from Sigma-Aldrich (St. Louis, MO) unless noted elsewhere.

### Cell culture model

Human, clear cell renal cell carcinoma cell lines, 786-O, 769-P, and Caki-1 were obtained from the American Type Culture Collection (ATCC—CRL-1932, CRL-1933, and HTB-46 respectively). All cell lines were authenticated and characterized as previously described by our group [34] and in accordance with recommendations to validate cell lines [35]. Cells used for this study were from the same stock and within passage limits recommended to minimize genetic drift. 786-O cells were cultured in Dulbecco's Modified Eagle Medium supplemented with 10% FBS and 1% ampicillin/1% Pen/Strep, 769-P cells were cultured in RPMI 1640 medium supplemented with 10% FBS and 1% Pen/Strep, and Caki-1 cells were cultured in Minimum Essential Medium supplemented with 10% FBS and 1% Pen/Strep at 37 °C in a 5% CO_2_ incubator. Cells were treated with varying concentrations (25, 50, 100, 250, 500, 750, 1000, or 2000 µM) of sodium oxalate (NaOx; soluble form of oxalate; cat#223,433) and CaOx crystals (insoluble form of oxalate) for 24 h. CaOx crystals were prepared as previously described [36], and were sonicated from a single stock before each experiment. All oxalate solutions were made fresh for each experiment and diluted to selected concentrations as indicated. In additional experiments, cells were exposed to oxalate (NaOx and CaOx crystals—100 and 750 µM) and rapamycin, a mTOR inhibitor (150 nM), for 24 h. Additional time points were not included for this pilot study to align all assays at a single time point.

### Cell proliferation and viability

The effect of oxalate on proliferation was determined using the Cell counting kit-8 (CCK-8) assay (cat#50–190–5565, Apexbio Technology LLC, Houston, TX). Cells were seeded at densities optimized for each line based on their proliferation rates and the linear detection range of the assay. Specifically, 786-O cells (10,000 cells/well), 769-P cells (10,000 cells/well), and Caki-1 cells (5,000 cells/well) were seeded in a clear 96-well plate and allowed to adhere overnight before treatment. Next, the cells were exposed to NaOx and CaOx crystals (25, 50, 100, 250, 500, 750, 1000, and 2000 µM) for 24 h. Following treatment, CCK-8 solution (10 µl) was added to each well before incubating the cells for 2 h in a 5% CO_2_ incubator at 37 °C. Following incubation, the plate was immediately read at 450 nm using a BioTek Synergy HT Microplate Reader (BioTek, Winooski, VT). Blanks were included to normalize to the background signal (cells with no CCK-8 reagent). Based on these experiments, the final concentrations of NaOx or CaOx crystals selected for the remaining experiments were 100 and 750 µM. As CCK‑8 measures metabolic activity rather than absolute cell counts, viability was measured using the Trypan Blue exclusion assay. In brief, cells were treated with NaOx or CaOx crystals (100 and 750 µM) as described above and subsequently diluted 1:1 with Trypan Blue and counted on a Bio-Rad TC20 Automated cell counter (Bio-Rad, Hercules, CA).

### Flow cytometry analyses

Flow cytometry was performed to evaluate cell cycle markers (PI staining) and apoptosis (Annexin V-PI staining). This assay is consistent with standard DNA-content based cell cycle methods, which quantify G1/S/G2/M distribution and do not distinguish G0 or cyclin status. 786-O cells (1 × 10^6^ cells/well) and 769-P cells (1 × 10^6^ cells/well) were seeded in 6-well plates and treated with NaOx and CaOx crystals (100 and 750 µM) for 24 h. Cells were trypsinized following treatment and washed with cold 1X PBS. For PI staining, cells were fixed with cold 4% paraformaldehyde (PFA) on ice for 10 min before being washed twice with cold 1X PBS. The cells were subsequently stained with 500 µl PI staining solution (FxCycle™ PI/RNase Staining Solution, cat#F10797, ThermoFisher Scientific, Waltham, MA) for 15 min at room temperature (RT) before analysis. For the apoptosis assay, cells were resuspended in Annexin-V binding buffer and stained with Annexin-V and PI for 15 min at RT (Dead Cell Apoptosis Kits with Annexin V for Flow Cytometry, cat #V13241, ThermoFisher Scientific, Waltham, MA). Next, the cells were resuspended in 1X Annexin-V binding buffer and kept on ice before analysis. Both PI and Annexin-V stainings were analyzed using a BD Biosciences LSRFortessa Flow cytometer (BD Biosciences, Franklin Lakes, NJ).

### RNA isolation and quantitative real time- polymerase chain reaction (qRT-PCR) analysis

qRT-PCR was used to assess the mRNA expression of genes related to mitochondrial metabolism and apoptosis. 786-O cells (300,000 cells/well) and 769-P cells (500,000 cells/well) were plated in 6-well plates, allowed to adhere overnight, and subsequently treated with NaOx and CaOx crystals (100 and 750 µM) for 24 h. Following treatment, RNA was isolated from cells using the GeneJET RNA Purification Kit (cat# K0731, ThermoFisher Scientific, Waltham, MA). RNA was treated with DNase I, RNase-free (cat#EN0521, ThermoFisher Scientific, Waltham, MA) to remove gDNA contamination. The cDNA was then synthesized using the Verso cDNA synthesis kit (cat#AB1453A, ThermoFisher Scientific, Waltham, MA). cDNA was used to perform qRT-PCR reactions using ABI PowerUP SYBR green master mix (cat#A25742, ThermoFisher Scientific, Waltham, MA). Human primers were designed using the Primer 3 software and synthesized from Integrated DNA technologies (Table 1). GAPDH was used as the housekeeping gene and the ΔΔCT method was used to quantify relative mRNA levels [37, 38].

**Table 1:** List of human primers used for quantitative real-time PCR analysis

Gene	Primer Sequence
Bcl-2-Associated Death Promoter, (BAD)	F 5’-CCCAGAGTTTGAGCCGAGTG −3’ R 5’-CCCATCCCTTCGTCGTCCT −3’
Bcl-2 Associated X, (BAX)	F 5’-CCCGAGAGGTCTTTTTCCGAG −3’ R 5’-CCAGCCCATGATGGTTCTGAT −3’
Succinate dehydrogenase (SDHA)	F 5’-GTTGCAAACAGGAACCCGAG −3’ R 5’-GTTCCCCAGAGCAGCATTGA −3’
Fumarate Hydratase (FH)	F 5’-CTGCGAGCTCATAGATTCTTGG −3’ R 5’-CTCTTGGGCAGGAATTTAGTGG −3’
Isocitrate dehydrogenase (IDH)	F 5’-CCAGGAGATCTTTGACAAGCAC −3’ R 5’-TGCACATCTCCGTCATAGTTCT −3’
Cytochrome c Oxidase Subunit I (COX I)	F 5’-CTGCTATAGTGGAGGCCGGA −3’ R 5’-GGGTGGGAGTAGTTCCCTGC −3’
Cytochrome c Oxidase Subunit IV (COX IV)	F 5’-CAGAGCCCTGGAACAAACTGGG −3’ R 5’-GACCTTCATTCTAAAGCAGCG −3’
Peroxisome proliferator-activated receptor gamma coactivator 1 alpha (PGC1-α)	F 5’-CCTGTGGATGAAGACGGATT −3’ R 5’-TAGCTGAGTGTTGGCTGGTG −3’
Transcription Factor A, Mitochondrial (TFAM)	F 5’- GTGGTTTTCATCTGTCTTGGCAAG −3’ R 5’- TTCCCTCCAACGCTGGGCAATT −3’

### Cellular bioenergetics

786-O cells (30,000 cells/well) and 769-P cells (35,000 cells/well) were plated in specialized Seahorse XF96-well plates. The cells were treated with NaOx or CaOx crystals (100 and 750 µM) for 24 h before measuring cellular bioenergetics using a “Mitochondrial Stress Test (MST)” (cat#103,015–100, Agilent Technologies, Santa Clara, CA) and the Seahorse XF96 Pro Analyzer (Agilent Technologies, Santa Clara, CA). The MST assay was performed by exposing the cells to oligomycin (1.5 µM), FCCP (1 µM), and Rotenone/Antimycin A (0.5 µM) to evaluate the amount of oxygen being consumed by the cells over time. In addition, the extracellular acidification rates (ECAR) were evaluated. Oligo-sensitive ECAR was calculated as the oligomycin response minus the baseline value, which was determined prior to oligomycin injection.

In additional experiments, the ATP Rate Assay Kit (cat#103,592–100, Agilent Technologies, Santa Clara, CA) was used to quantify mitochondrial and glycolytic ATP production based on oligomycin‑sensitive oxygen consumption and glycolytic proton efflux. Cells were exposed to oligomycin (1.5 µM) and Rotenone/Antimycin A (0.5 µM) over time to determine ATP production rates. This assay does not incorporate FCCP and does not measure maximal respiration. In additional experiments, 786-O and Caki-1 cells (20,000 cells/well) were plated and exposed to oxalate with or without rapamycin (150 nM) for 24 h before measuring cellular bioenergetics and ATP production rates as described above. At the end of all assays, the media was removed and the cells were lysed with RIPA buffer (ThermoFisher Scientific, Waltham, MA) before storing the plate at −20 °C. A BCA Protein assay was later performed to measure the protein concentrations per well. All data were normalized to the total amount of protein per well.

### Mitochondrial and oxidative stress fluorescence assays

Relative Mitochondrial activity and oxidative stress levels were assessed in separate experiments using MitoTracker Red CMXRos (cat#M46752, ThermoFisher Scientific, Waltham, MA) and H2-DCFDA (2’, 7’-dichlorodihydrofluoroscein diacetate; cat#D399, ThermoFisher Scientific, Waltham, MA) fluorescence probes, respectively. MitoTracker Red CMXRos fluorescence labels mitochondria with intact membrane potential, and H2-DCFDA fluorescence serves as a direct indicator of total ROS levels in live cells. 786-O cells (10,000 cells/well) and 769-P cells (10,000 cells/well) were seeded in black 96-well plates and allowed to adhere to the plate overnight. Cells were subsequently exposed to NaOx and CaOx crystals (100 and 750 µM) for 24 h. In additional experiments, 786-O and Caki-1 cells (10,000 cells/well) were plated and exposed to oxalate with or without rapamycin (150 nM) for 24 h. Hydrogen peroxide (0.3%, 30 min) was included as a positive control for H2-DCFDA experiments. Following treatment, the media was aspirated, and the cells were washed once with Hanks' Balanced Salt Solution (HBSS). Cells were stained separately with MitoTracker Red CMXRos (500 nM) and H2-DCFDA (10 µM) at 37 °C in a 5% CO_2_ incubator for 30 min. The plates were immediately read in a BioTek Synergy HT Microplate Reader. A background control (cells without staining) was included for all assays to determine the mean fluorescence values.

### Repair assisted damage detection (RADD) assay

The effect of oxalate on DNA damage in RCC cells treated with NaOx or CaOx crystals was determined using a RADD assay, which can identify genomic DNA with oxidative lesions, crosslinks, alkylation, uracil, and abasic sites [39, 40]. In brief, this assay consists of incubating cells with a cocktail of bacterial glycosylases [(Fapy-DNA glycosylase (FPG), Endonuclease IV (Endo IV), Endonuclease VIII (Endo VIII), T4 pyrimidine dimer glycosylase (T4PDG), and uracil DNA glycosylase (UDG), and the human 3-alkyladenine DNA glycosylase (AAG))] specific for identifying and removing several DNA lesion types. The remaining gaps or strand breaks are then labeled with a digoxigenin-labeled dUTP inserted with Klenow exo-, which lacks proofreading. 786-O cells (1 × 10^6^ cells/well) and 769-P cells (1 × 10^6^ cells/well) were plated in glass-bottomed fluorodishes and kept overnight in a 5% CO_2_ incubator at 37 °C. The next day, cells were treated with oxalate (100 and 750 µM) for 24 h. Cells were then washed twice with 1X PBS before being fixed with 4% PFA for 10 min at RT and washing the cells three times with 1X PBS.

Next, cells were permeabilized with Biotium permeabilization buffer (22,016, Fremont, CA, USA) with 0.05% Triton-X for 10 min at 37 °C. The cells were subsequently washed three times with 1X PBS before being exposed to the lesion removal cocktail, FPG (4U, cat#M0240, New England BioLabs (NEB), Ipswich, MA, USA,), T4 PDG (5U, cat#M0308, NEB), UDG (2.5U, cat#M0280, NEB), EndoIV (5U, cat#M0304, NEB), EndoVIII (5U, cat#M0299, NEB), AAG (5U, cat#M0313, NEB) resuspended in 1X ThermPol buffer (cat#B9004S, NEB) + 200 µg/ml BSA (cat#B4287-25G, Sigma Aldrich, St. Louis, MO, USA) for 1 h at 37 °C in a hybridization Fluorescent In-Situ Hybridization oven [41]. Next, the gap-filling mixture of Klenow exo- and digoxigenin-labeled dUTP (cat#11,093,088,910, Sigma Aldrich, St. Louis, MO, USA) in 1X ThermPol buffer + BSA was added to the cells and incubated again at 37 °C for 1 h. The cells were washed three times with 1X PBS and blocked with 2% BSA (cat#001–000–162, Jackson ImmunoResearch) in PBS for 30 min. After blocking, the samples were incubated with anti-digoxigenin (1:250; cat#ab420, Abcam) or anti-mouse IgG1 isotype control (1:625; cat#5415, Cell Signaling) primary antibodies for 1 h at RT.

Next, the cells were washed three times with 1X PBS and incubated for 1 h with a secondary anti-rabbit Alexa Fluor 546 (1:400; cat#A11010, ThermoFisher Scientific, Waltham, MA) antibody for 1 h at RT. Hoechst solution (1:800; cat#H3570, ThermoFisher Scientific, Waltham, MA) was added to the cells 10 min before the end of the incubation period to stain nuclei. Cells were subsequently washed three times with 1X PBS and mounted with 18 mm coverglass with Prolong Gold (cat#P36930, ThermoFisher Scientific, Waltham, MA). An all-in-one fluorescence Keyence microscope (cat#BZ-X800, Keyence, Osaka, Japan) using the 20X (NA 0.75) objective was used for all imaging. At least 100 cells were imaged over three biological replicates for each experimental group. The Nikon Elements software was used to define the region of interest (ROI) for the nucleus, and the fluorescent intensity for each ROI was calculated. GraphPad Prism was used to identify outliers using the ROUT 1% method, and the remaining data was shown as the mean fluorescent intensity of the nucleus.

### Western blotting

786-O (750,000 cells/well) and Caki-1 (1 million cells/well) cells were plated in 6-well dishes. The following day, cells were treated with oxalate and 150 nM rapamycin or vehicle control. After 24 h, the cells were harvested and lysed with Pierce RIPA buffer (ThermoFisher Scientific, Waltham, MA) containing Pierce protease and phosphatase inhibitors (ThermoFisher Scientific, Waltham, MA). The total protein concentration was determined using a Pierce BCA protein assay (ThermoFisher Scientific, Waltham, MA). Cell lysates (30 μg) were resolved by SDS-PAGE on a 4–15% Tris–glycine (TGX) gel (Bio-Rad, Hercules, CA), and transferred to a PVDF membrane (Bio-Rad, Hercules, CA) using a Trans-blot Turbo (Bio-Rad, Hercules, CA). The membrane was blocked with 5% Blotting Grade Blocker (Bio-Rad, Hercules, CA) in PBST and probed overnight with primary antibody against phospho-mTOR (Cell Signaling, Danvers, MA, Cat. #5536), phospho-p70S6K (Cell Signaling, Danvers, MA, Cat. #9234), and actin (Santa Cruz Biotechnology, Dallas, TX, Cat. #sc-47778). The next day, the membranes were probed with Horse Radish Peroxidase conjugated secondary antibodies and visualized using Clarity Max (Bio-Rad, Hercules, CA) on an ImageQuant LAS 4000 imager and software (GE Healthcare Life Sciences, Marlborough, MA). The membranes were then incubated in Restore Western Stripping buffer (ThermoFisher Scientific, Waltham, MA) according to the manufacturer’s recommendation. The membranes were re-probed overnight with antibodies against mTOR (Cell Signaling Danvers, MA, Cat. # 2983) and p70S6K (Cell Signaling, Danvers, MA Cat. #34,475) and reimaged as described above.

### Statistical analyses

All experiments were performed 3 times using independent biological replicates, and multiple technical replicates were averaged within each biological replicate. GraphPad Prism (version 10.0.0 for Windows, GraphPad Software, Boston, MA) was used to perform the statistical analyses and to generate graphs. The data are represented as mean ± SEM for 3 experiments unless stated. Differences in the groups were determined using a one-way ANOVA followed by Tukey's multiple comparisons test or Dunnett’s post hoc test. For cell cycle and apoptosis, statistical analysis was done using two-way ANOVA with Tukey’s post hoc. All statistical tests were two-sided and p < 0.05 was considered statistically significant. Numerical trends that did not reach statistical significance reflected variability among groups rather than the absence of statistical testing.

## Results

### The effect of oxalate on cell proliferation, cell cycle, and cell death in ccRCC

**Fig. 1:**
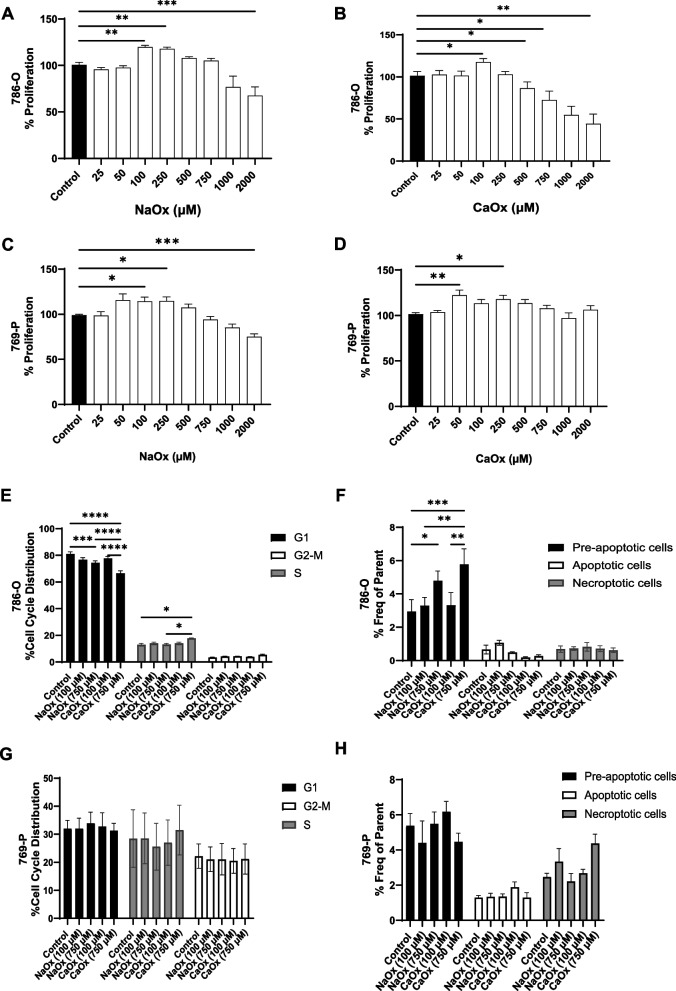
The effect of oxalate treatment on cell proliferation, cell cycle, and apoptosis in ccRCC. **A**, **B** 786-O cells and **C**, **D** 769-P cells were exposed to various NaOx and CaOx concentrations (25–2000 µM) for 24 h to assess cell proliferation using a CCK-8 assay. Flow cytometry was used to assess cell cycle distribution and apoptosis in 786-O cells (**E** and **F**, respectively) and 769-P cells (**G** and **H**, respectively). All experiments were repeated at least three times. Data are represented as mean ± SEM. * *p* < 0.05; ** *p* < 0.01; *** *p* < 0.001; **** *p* < 0.0001

To initially assess the effects of oxalate exposure (soluble—NaOx and insoluble—CaOx crystals) on 786-O and 769-P cells, we performed the CCK-8 assay using a range of concentrations (25–2000 µM). As shown in Fig. 1A, NaOx exposure (100 and 250 µM) significantly increased proliferation in 786-O cells. However, proliferation was significantly reduced when cells were exposed to higher concentrations of NaOx (Fig. 1A). When 786-O cells were treated with CaOx crystals, there was a significant increase in proliferation at 100 µM (Fig. 1B). In addition, proliferation was significantly reduced in a dose-dependent manner when cells were exposed to higher CaOx crystal concentrations (Fig. 1B). Next, we performed the Trypan Blue exclusion assay and determined that 100 µM of NaOx increased viability; whereas, 750 µM of CaOx crystals decreased viability in 786-O cells (Supplementary Fig. 1 A). In regard to 769-P cells, proliferation was significantly increased with 100 and 250 µM of NaOx and started to decline with exposure to higher concentrations of NaOx (Fig. 1C). 769-P cells exposed to CaOx crystals had a significant increase in proliferation at 50 and 250 µM though higher concentrations did not alter cell proliferation (Fig. 1D) but did decrease cell viability (Supplementary Fig. 1B). These data demonstrate that specific oxalate concentrations (100–250 µM) increase cell proliferation in ccRCC cells. Based on these data, we opted to assess the effect of both NaOx and CaOx crystals at 100 and 750 µM for the remaining experiments.

An increase in proliferation in these cells could be due to alterations in the cell cycle or the rate of apoptosis. Flow cytometry was used to assess the cell cycle using propidium iodide (PI) staining and apoptosis using Annexin V/PI staining. The cell cycle consists of several phases, including the G1 phase (cell growth and preparation for DNA replication), the S phase (DNA replication), and the G2-M phase (vital for cell growth, DNA integrity and initiating mitosis). We observed a significant decrease in the percentage of 786-O cells in the G1 phase when exposed to NaOx or CaOx crystals at 750 µM compared to the control cells (Fig. 1E). Concurrently, 786-O cells exposed to CaOx crystals (750 µM) had a significant increase in the percentage of cells in the S phase (Fig. 1E). There was no difference in the percentage of cells in the G2-M phase among all of the treatment groups (Fig. 1E). 786-O cells also exhibited a significant increase in the percentage of pre-apoptotic cells when exposed to higher concentrations of oxalate (750 µM) (Fig. 1F). In contrast, we did not observe any differences in both apoptotic and necroptotic cells with oxalate exposure in 786-O cells (Fig. 1F). Cell cycle and cell death were not altered in 769-P cells exposed to NaOx or CaOx crystals at any of the selected concentrations (Fig. 1G and H, respectively). Collectively, these data suggest there are inherent differences in the ccRCC cell lines and that reduced proliferation in 786-O cells at higher oxalate concentrations could be due to increased early stress signals.

### The effect of oxalate on cell death and mitochondrial mRNA levels in ccRCC

**Fig. 2:**
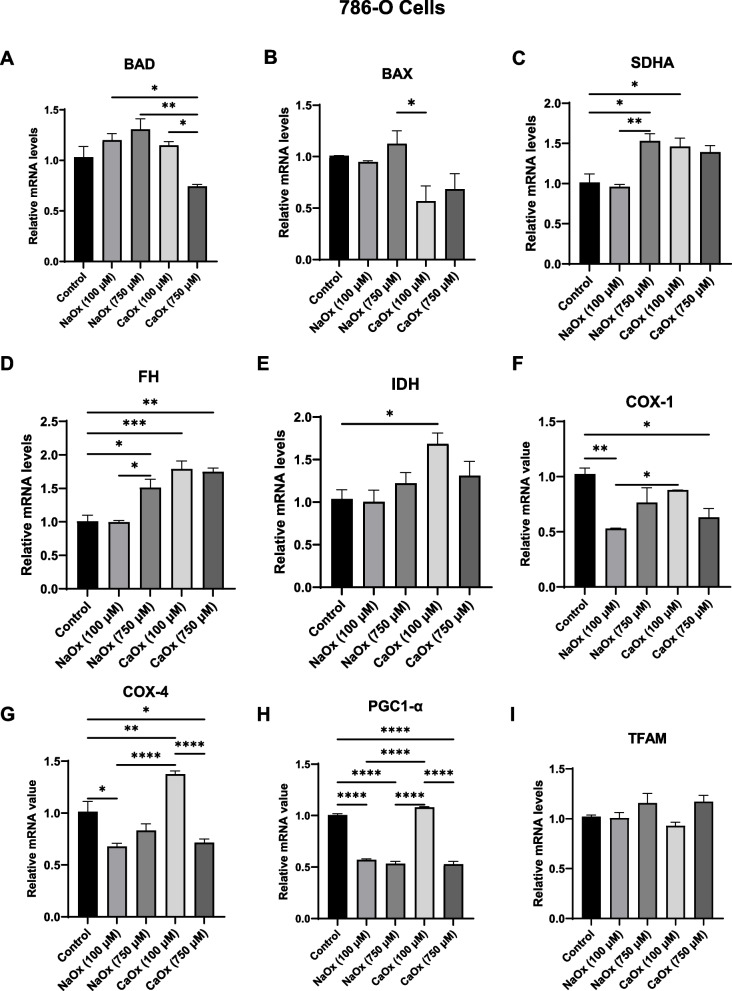
The effect of oxalate treatment on cell death and mitochondrial gene expression in 786-O cells. Relative mRNA levels were assessed in 786-O cells following 24 h oxalate exposure using quantitative Real-Time PCR (qRT-PCR). Bar graphs show apoptosis markers—(**A**) BAD, (**B**) BAX, and mitochondrial markers—(**C**) Succinate dehydrogenase (SDHA), (**D**) Fumarate hydratase (FH), (**E**) Isocitrate dehydrogenase (IDH), (**F**) COX-1, (**G**) COX-4, and upstream mitochondrial regulators (**H**) PGC-1α and (**I**) TFAM relative mRNA levels. All experiments were repeated at least three times. Data are represented as mean ± SEM. * *p* < 0.05; ** *p* < 0.01; *** *p* < 0.001; **** *p* < 0.0001

To assess whether oxalate influences apoptosis, we measured the mRNA levels of the pro-apoptotic genes, Bcl-2-Associated Death Promoter (*BAD*) and Bcl-2 Associated X (*BAX*) in 786-O cells. Both *BAD* and *BAX* mRNA levels were not altered compared to control cells (Fig. 2A-B). We next examined mitochondrial metabolic gene expression by quantifying transcripts involved in the TCA cycle, electron transport chain (ETC), and mitochondrial regulation. Among TCA cycle genes, succinate dehydrogenase (*SDHA*) was significantly increased in 786‑O cells treated with 750 µM NaOx and 100 µM CaOx (Fig. 2C). Fumarate Hydratase (*FH*) expression was significantly elevated following 750 µM NaOx and both 100 µM and 750 µM CaOx treatments (Fig. 2D). Isocitrate dehydrogenase (*IDH*) expression increased only in response to 100 µM CaOx crystals (Fig. 2E).

For ETC components, Cytochrome c Oxidase Subunits I and IV (*COX1*, *COX4*) expression decreased with 100 µM NaOx (Fig. 2F–G). In contrast, 100 µM CaOx crystals significantly increased *COX4* levels, while higher CaOx exposure reduced both *COX1* and *COX4* expression (Fig. 2F–G). Lastly, we assessed upstream mitochondrial biogenesis regulators. Peroxisome proliferator-activated receptor gamma coactivator 1 alpha (*PPARGC1A*) mRNA levels were significantly decreased in response to 100 and 750 µM NaOx and 750 µM CaOx (Fig. 2H). Transcription Factor A, Mitochondrial (*TFAM*) mRNA levels were not modified in response to oxalate (Fig. 2I). These data suggest that oxalate alters metabolic transcripts in a dose‑dependent manner in 786‑O cells.

**Fig. 3:**
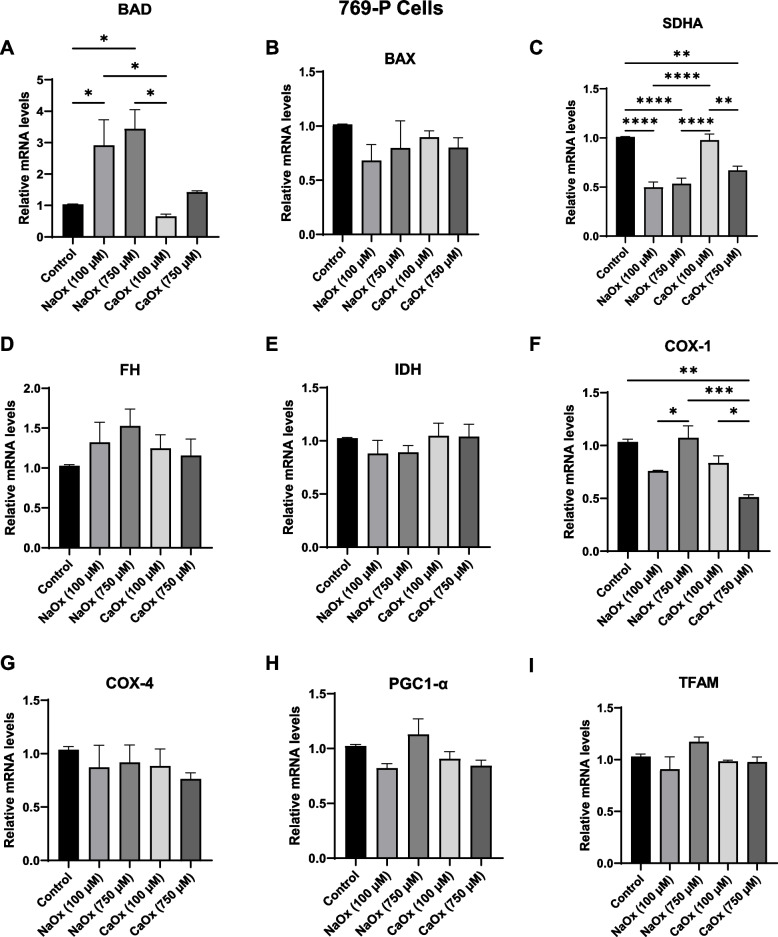
The effect of oxalate treatment on cell death and mitochondrial gene expression in 769-P cells. Relative mRNA levels were assessed in 769-P cells following oxalate exposure (24 h) using quantitative Real-Time PCR (qRT-PCR). Bar graphs show apoptosis markers—show (**A**) BAD, **B** BAX), and mitochondrial markers—**C** Succinate dehydrogenase (SDHA), **D** Fumarate hydratase (FH), **E** Isocitrate dehydrogenase (IDH), **F** Cytochrome c oxidase subunit I (COX-1), **G** Cytochrome c oxidase subunit 4 (COX-4), and upstream mitochondrial regulators (**H**) PGC-1α and **I** TFAM relative mRNA levels. All experiments were repeated at least three times. Data are represented as mean ± SEM. * *p* < 0.05; ** *p* < 0.01; *** *p* < 0.001; **** *p* < 0.0001

In 769-P cells, *BAD* expression was only increased in cells exposed to 100 and 750 µM NaOx and not with CaOx crystal treatment (Fig. 3A). *BAX* expression was similar to control cells following oxalate exposure (Fig. 3B). *SDHA* and *COX1* were the only genes significantly altered by oxalate treatment (Fig. 3C and F). Specifically, 100 and 750 µM NaOx and 750 µM CaOx reduced *SDHA* mRNA levels in 769-P cells compared to the control cells (Fig. 3C). This is in contrast to the 786-O cell line where select oxalate conditions led to increased *SDHA* expression. *COX1* gene expression was only decreased in cells treated with 750 µM CaOx crystals (Fig. 3F). *FH*, *IDH*, *COX4*, *PPARGC1A* and *TFAM* mRNA levels were not different in 769-P cells with oxalate treatment (Fig. 3D, E, G, H and I). These findings demonstrate that 769‑P cells display minimal transcriptional changes with oxalate exposure.

### The effect of oxalate on mitochondrial metabolism in ccRCC

**Fig. 4:**
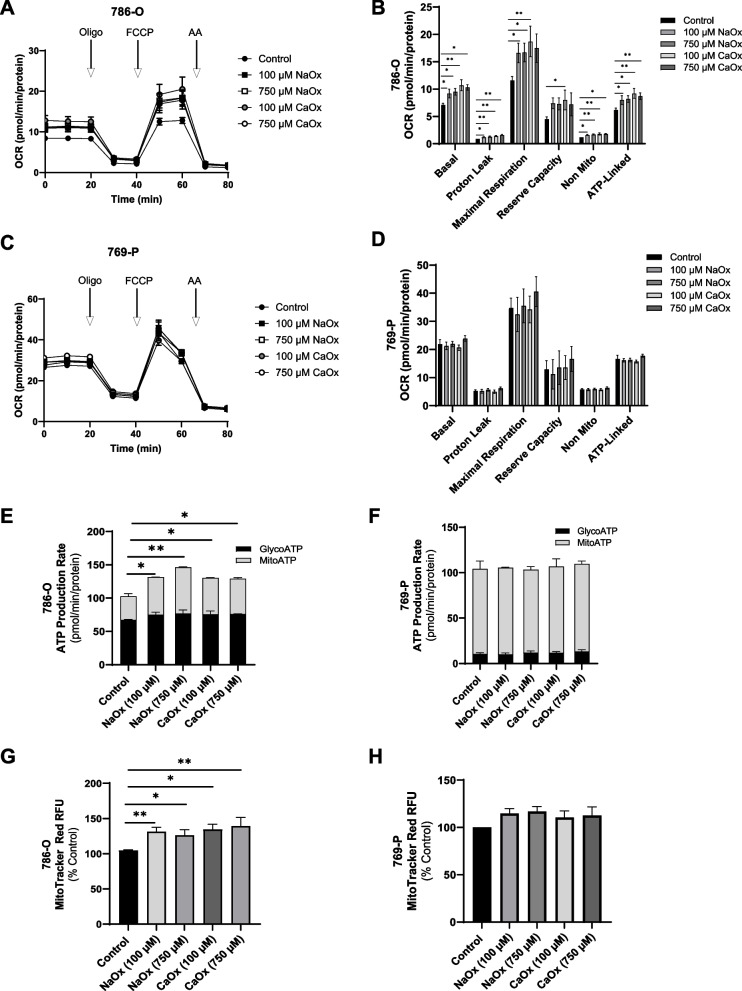
The effect of oxalate treatment on metabolism and ATP levels in ccRCC. 786-O cells and 769-P cells were exposed to NaOx and CaOx (100 and 750 µM) for 24 h. **A** The oxygen consumption rate (OCR) and **B** individual OCR parameters for 786-O cells were determined using the Seahorse Mitochondrial Stress Test. The (**C**) OCR and **D** individual OCR parameters for 769-P cells are also shown. ATP Rates were calculated using the Seahorse ATP Rate Assay Kit for (**E**) 786-O and **F** 769-P cells. MitoTracker Red fluorescence intensity of (**G**) 786-O cells and **H** 769-P cells are shown. All experiments were repeated at least three times with 5–6 replicates per group. Data are represented as mean ± SEM. * *p* < 0.05; ** *p* < 0.01

We further analyzed the effects of oxalate exposure on metabolism in both cell lines. As shown in Fig. 4A, the Seahorse Mitochondrial Stress Test demonstrated that both NaOx and CaOx crystals (100 and 750 µM) increased the oxygen consumption rate (OCR) of 786-O cells compared to cells not exposed to oxalate. In particular, basal respiration, proton leak, maximal respiration, non-mitochondrial respiration, and ATP production were significantly elevated (Fig. 4B). Reserve capacity was significantly increased in cells treated with CaOx crystals (100 µM) (Fig. 4B). To further characterize the effect of oxalate on energy production, we assessed the ECAR in 786-O cells (Supplementary Fig. 2 A). Both NaOx and CaOx crystals (both 100 and 750 µM) significantly increased oligo-sensitive ECAR compared to control cells (Supplementary Fig. 2B), confirming these cells are metabolically reprogramming. Regarding 769-P cells, OCR (Fig. 4C and D) was not impacted by any of the oxalate treatment groups. In addition, only the highest concentration of CaOx crystals (750 µM) impacted the ECAR (Supplementary Fig. 2 C) and increased oligo-sensitive ECAR compared to control cells (Supplementary Fig. 2D). We next evaluated ATP production rates in 786-O cells and determined that both NaOx and CaOx crystals significantly increased total ATP production compared to cells not exposed to oxalate (Fig. 4E). In contrast, ATP production rates were not different in 769-P cells (Fig. 4F). Lastly, oxalate significantly increased the number of active mitochondria in 786-O cells (Fig. 4G); whereas, it had no effect on 769-P cells (Fig. 4H).

### The effect of oxalate on oxidative stress and DNA damage in ccRCC

**Fig. 5:**
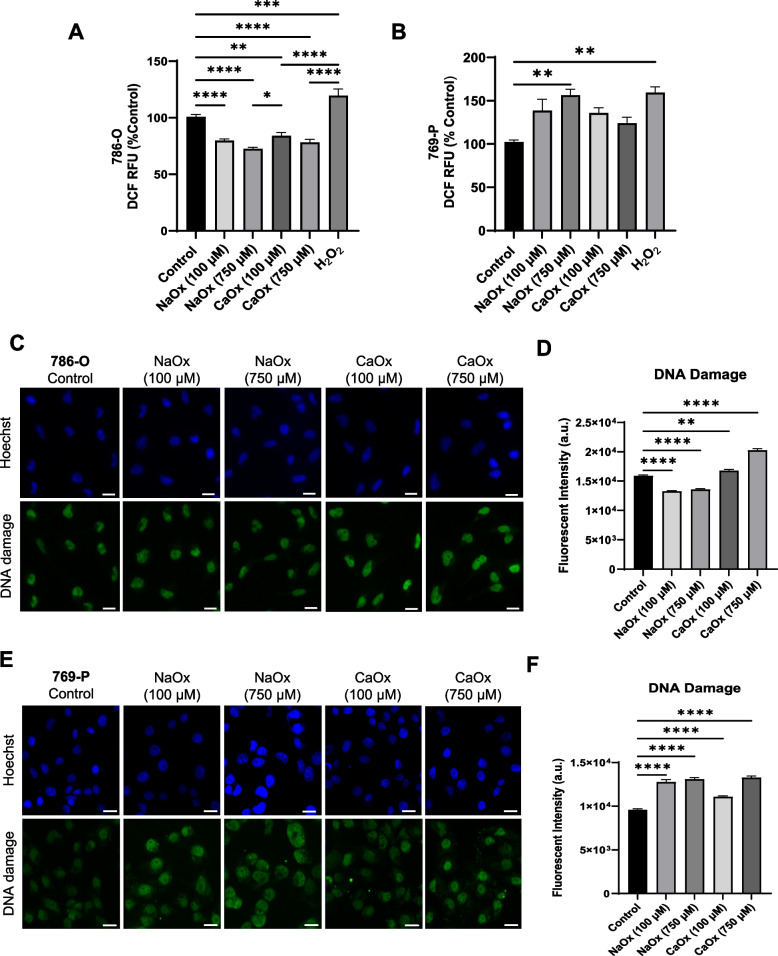
The effect of oxalate treatment on oxidative stress and DNA damage in ccRCC. 786-O cells and 769-P cells were exposed to NaOx and CaOx (100 and 750 µM) for 24 h. Total cellular ROS levels in (**A**) 786-O cells and (**B**) 769-P cells were determined using a H2-DCFDA fluorescence assay. Representative images and quantification of DNA adducts in (**C**, **D**) 786-O cells and (**E**, **F**) 769-P cells using the Repair Assisted Damage Detection (RADD) assay. Scale bar, 50 µM. Quantification of the images were determined for at least 100 cells per biological replicate. All experiments were repeated at least three times. Data are represented as mean ± SEM. * *p* < 0.05; ** *p* < 0.01; *** *p* < 0.001; **** *p* < 0.0001

To determine the effect of oxalate on oxidative stress in ccRCC, we measured total reactive oxygen species (ROS) levels using the H2-DCFDA fluorescence assay. It is well established that lower amounts of ROS can stimulate cellular proliferation and are essential for cell signaling; whereas, higher levels of ROS can cause oxidative stress and DNA damage to the cell [3]. Both NaOx and CaOx crystals caused a significant reduction in total ROS levels in 786-O cells compared to control cells (Fig. 5A). Interestingly, 769-P cells exposed to 750 µM NaOx had an increase in ROS production compared to the other treatment groups (Fig. 5B). Cells treated with the positive control, hydrogen peroxide (H_2_O_2_) significantly increased ROS generation as expected in both cell lines (Fig. 5A and B). In addition, it was determined that DNA damage was significantly reduced in 786-O cells treated with NaOx (Fig. 5C and D). In contrast, 786-O cells treated with CaOx crystals had a significant increase in DNA damage compared to cells not treated with oxalate (Fig. 5C and D). Regarding 769-P cells, both NaOx and CaOx crystals caused a significant increase in DNA damage, which could be a result of increased ROS production (Fig. 5E and F).

### The effect of oxalate on Caki-1 cells and mTOR signaling in ccRCC

To confirm if these findings were ccB subtype and mTOR dependent, we used Caki-1 cells (ccRCC, ccB subtype) and rapamycin, an established mTOR inhibitor. We determined that NaOx (25–1000 µM) and CaOx crystals (25–2000 µM) did not affect the proliferation of Caki-1 cells (Supplementary Fig. 3 A and 3B). Only the highest NaOx concentration (2000 µM) reduced proliferation (Supplementary Fig. 3 A). Additionally, cells treated with higher concentrations of NaOx and CaOx crystals had reduced cell viability (Supplementary Fig. 3C).

We also determined that rapamycin alone did not affect proliferation in either cell line (Supplementary Fig. 4 A and 4B). A concentration of 150 nM rapamycin was used because nanomolar doses are routinely applied in RCC cell studies to inhibit mTORC1 signaling while maintaining cell viability [42–44]. We next evaluated the effect of rapamycin on mTOR protein expression following oxalate exposure. The hallmark of mTOR activation is phosphorylation of its downstream target S6 kinase (S6K), which can phosphorylate mTOR and modulate its activity [45]. As shown in Supplementary Fig. 4 C, both 786-O and Caki-1 control cells express p-mTOR and p-S6K protein. However, these levels were not further altered with oxalate treatment. We further determined that p-mTOR and p-S6K levels were inhibited with rapamycin treatment (Supplementary Fig. 4 C).

### The effect of inhibiting mTOR signaling on metabolism and oxidative stress in ccRCC

**Fig. 6:**
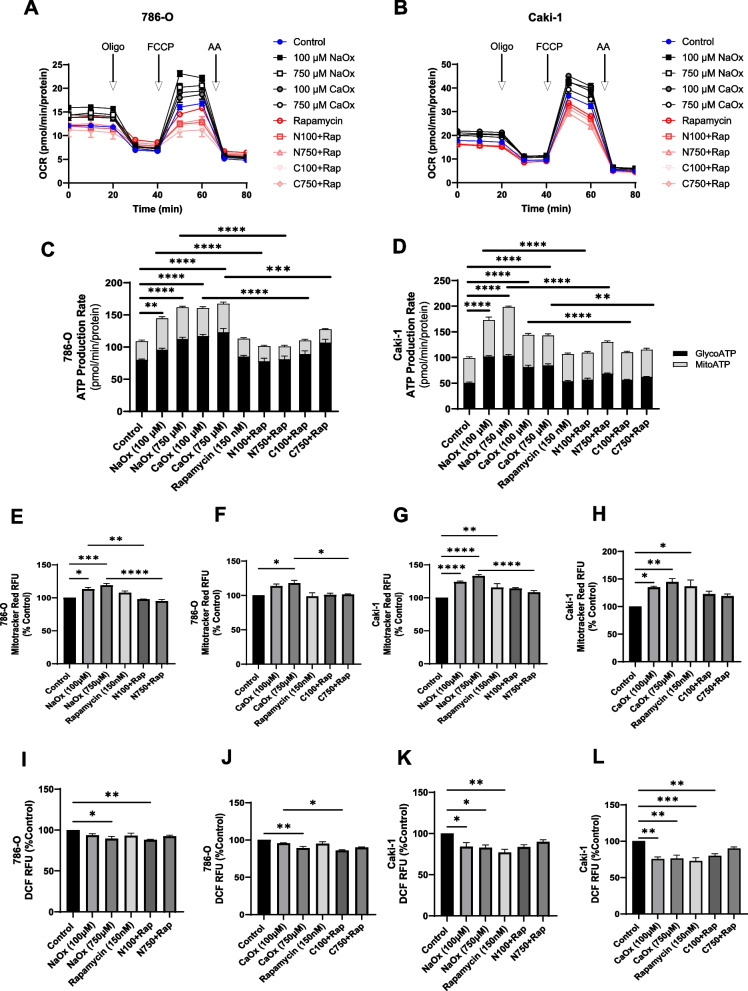
The effect of rapamycin on metabolism and ATP levels in ccRCC treated with oxalate. 786-O and 769-P cells were exposed to NaOx and CaOx (100 and 750 µM) with or without rapamycin (150 nM) for 24 h. The oxygen consumption rate (OCR) for (**A**) 786-O and (**B**) Caki-1 cells were determined using the Seahorse Mitochondrial Stress Test. ATP Rates were calculated using the Seahorse ATP Rate Assay Kit for (**C**) 786-O and (**D**) Caki-1 cells. MitoTracker Red fluorescence intensity of (**E**–**F**) 786-O cells and (**G**-**H**) Caki-1 cells are shown. Total cellular ROS levels were also determined in (**I**-**J**) 786-O cells and (**K**-**L**) Caki-1 cells using a H2-DCFDA fluorescence assay. All experiments were repeated at least three times with 5–6 replicates per group. Data are represented as mean ± SEM. * *p* < 0.05; ** *p* < 0.01; *** *p* < 0.001; **** *p* < 0.0001

Next, we investigated the effect of rapamycin on metabolism in 786-O and Caki-1 cells. Both 100 and 750 µM concentrations of NaOx and CaOx crystals significantly increased mitochondrial respiration in 786-O cells (Fig. 6A). When 786-O cells were treated with oxalate (NaOx and CaOx crystals) and rapamycin, OCR were similar or lower than control cells (Fig. 6A). Rapamycin restored basal, ATP-linked, maximal respiration, and reserve capacity rates to control cell levels (Supplementary Fig. 5A-B, 5D-5E) but did not affect proton leak (Supplementary Fig. 5C). Notably, rapamycin increased non-mitochondrial OCR in 786-O cells alone and with CaOx crystals (100 µM) (Supplementary Fig. 5F). Regarding Caki-1 cells, oxalate increased OCR and individual parameters in a dose dependent manner and this was prevented with rapamycin exposure (Fig. 6B and Supplementary Fig. 6A-6F).

In addition to evaluating mitochondrial respiration, we assessed ECAR. NaOx (100 and 750 µM) induced ECAR in 786-O cells; whereas, CaOx crystals had no effect (Supplementary Fig. 7A and 7B). When cells were co-treated with oxalate and rapamycin, ECAR levels were significantly reduced compared to cells treated with oxalate alone. CaOx crystals did not induce ECAR in these cells. In the case of the Caki-1 cells, only 100 µM of CaOx crystals significantly induced ECAR (Supplementary Fig. 7 C and 7D). NaOx had no effect on these cells. However, rapamycin significantly reduced ECAR in all cells treated with NaOx or CaOx crystals (Supplementary Fig. 7 C and 7D). Next, we assessed ATP production rates and determined that both 786-O and Caki-1 cells produce more ATP when treated with oxalate and this was inhibited with rapamycin (Fig. 6C and D). The primary source of ATP was from glycolysis in 786-O cells (Fig. 6C); whereas, ATP was generated from both mitochondrial and glycolytic sources in Caki-1 cells (Fig. 6D).

We further determined that rapamycin reduced NaOx-induced MitoTracker Red fluorescence intensity in 786-O cells (Fig. 6E) and Caki-1 cells (Fig. 6G) to levels detected in control cells. Only 750 µM CaOx crystals induced MitoTracker Red in 786-O cells and this was reduced with rapamycin (Fig. 6F). In contrast, Caki-1 cells had increased signal with both concentrations of CaOx crystals. However, rapamycin did not significantly alter the number of active mitochondria (Fig. 6H). Lastly, we assessed oxidative stress using H2-DCFDA staining. Consistent with our findings above, oxalate decreased ROS levels in 786-O cells and co-treatment with rapamycin further decreased ROS levels in 786-O cells (Fig. 6I and J). Both NaOx and CaOx crystals caused a significant decrease in ROS levels in Caki-1 cells and rapamycin did not modify ROS levels (Fig. 6K and L).

## Discussion

Clinical and experimental studies have suggested an association between RCC and KS disease [22–25, 27]. A potential link between these two pathologies is oxalate, which is found in 80% of KS and several plant-derived foods. CaOx crystals have been found in tumors from patients with renal cancer [46, 47]. In addition, oxalate induces breast cancer cell proliferation and carcinogenic properties in a human renal epithelial cell line [28, 48]. However, the physiological relevance of oxalate on RCC is unclear. In this pilot study, we examined how oxalate alters metabolic and molecular pathways in ccRCC. Our findings suggest a complex interplay between metabolic reprogramming, oxidative stress, and DNA damage, which offers new insight into the potential association of ccRCC and KS disease.

We used two well-established ccRCC human cell lines, 786-O and 769-P for these studies. 786-O cells are commonly used in RCC research as they display many hallmarks of ccRCC, including the mutation of the *VHL* gene, a key tumor suppressor [49]. VHL is important for targeting and degrading proteins such as hypoxia-inducible factor (HIF). When VHL is mutated, this leads to the accumulation of HIF and activation of genes involved in angiogenesis, cell proliferation, and metabolism. 769-P cells are also defective in *VHL* expression and have side populations that exhibit tumorigenicity, self-renewal, and are resistant to chemotherapy [49, 50]. It has been established that 786-O cells can promote bone metastasis *in vivo*, which is a major metastatic site of RCC [51]. In contrast, 769-P cells have reduced tumorigenicity in mice compared to other RCC cell lines [50]. Using both cell line models allowed us to examine whether oxalate produces cell-line–specific phenotypes. Indeed, our findings show that oxalate enhances proliferation and metabolism in 786-O cells but does not elicit similar responses in 769-P cells.

We first evaluated how soluble and insoluble oxalate affects proliferation, viability, cell cycle, and apoptosis. The concentrations used here align with prior studies that have examined the effects of oxalate (0.2 to 2 mM) on renal epithelial cells [12, 52] and are physiological relevant concentrations of urinary oxalate (0.1 to 5 mM) in healthy individuals and patients with primary hyperoxaluria [53]. In 786-O cells, low concentrations of oxalate increased cell proliferation; whereas, higher concentrations decreased viability. This is consistent with previous reports that established that higher oxalate concentrations can compromise the viability of renal epithelial cells through mechanisms involving membrane permeability and oxidative stress [54, 55]. Such biphasic responses are typical in cells exposed to low or high concentrations of a stressor [56], suggesting that lower concentrations of oxalate could stimulate survival or adaptation in 786-O cells, while higher concentrations could suppress growth. Notably, cell viability was not altered in these cells regardless of the oxalate concentrations. In 769-P cells, NaOx reduced proliferation in a concentration dependent manner. In contrast, higher concentrations of CaOx crystals reduced cell viability and did not impact proliferation.

Based on the proliferation data in 786-O cells, we hypothesized that oxalate may influence proliferation via changes in the cell cycle and apoptosis. Oxalate has been shown to induce proliferation in renal epithelial [57] and breast cancer cells [28]. Flow cytometry analysis revealed that oxalate significantly reduced the number of cells in the G1 phase, suggesting enhanced cell cycle entry and potentially DNA damage. Conversely, oxalate did not alter the cell cycle in 769-P cells, which is consistent with their minimal proliferation rates.

The cell cycle and apoptosis are interconnected processes that determine a cell's fate. Oxalate is reported to induce apoptosis in human renal tubular epithelial cells at high concentrations (i.e. 5 mM) [58] and to induce apoptosis and necrosis simultaneously in a monkey renal epithelial cell line [59]. In our study, higher oxalate concentrations of NaOx and CaOx (750 µM) induced early apoptotic signaling in 786-O cells but did not promote late apoptosis or necrosis. Notably, both *BAD* and *BAX* gene expression, which are key players in apoptosis, were not different in these cells compared to control cells*.* In contrast, 769-P cells exposed to both concentrations of NaOx had increased *BAD* levels compared to control cells, suggesting that the cells were shifting toward more apoptotic signaling, while BAX levels were not different. Indeed, the highest concentration of oxalate did promote some cell death in 769-P cells.

Collectively, these findings suggest that oxalate increases the proliferative potential of 786-O cells, especially at lower doses, and higher doses of oxalate tend to result in pre-apoptosis. Importantly, BrdU incorporation and caspase activation would help to refine these interpretations. The differences between our *BAD/BAX* mRNA and flow-cytometry apoptosis data suggest that post-translational modifications may be occurring. However, we did not assess protein levels as this was outside the scope of the study and will be pursued in the future. Based on these findings, we postulated that oxalate may promote metabolic shifts in ccRCC to fuel the changing energy demands of the cells.

Mitochondria modulate metabolism and play an important role in cancer progression and immune responses [60]. Oxalate was previously demonstrated to reduce mitochondrial function in immune cells [16–19]. Further, oxalate was reported to stimulate mitochondrial superoxide generation in renal epithelial cells [61] and mitochondrial permeability transition in isolated rat kidney mitochondria [62]. In our study, oxalate differentially impacted several mitochondrial genes in 786-O and 769-P cells. Specifically, key metabolic genes involved in the TCA cycle (*SDHA*, *FH*, *IDH*) were upregulated in 786-O cells treated with oxalate, suggesting that the cells are attempting to maintain metabolic function under oxalate-mediated stress. Regarding 769-P cells, oxalate reduced *SDHA* and *COX1* mRNA levels, implying a distinct metabolic response. These observations show oxalate increases proliferation, early stress signaling, and metabolism in 786-O cells while not significantly impacting 769-P cells.

To determine whether metabolic shifts were occurring in ccRCC, we evaluated cellular bioenergetics and redox status. The “Warburg Effect” is a well-established concept that describes how cancer cells suppress mitochondrial respiration and favor glycolysis for energy production. During this process, glucose is converted to lactate through glycolysis and cultivates an environment for tumor growth, invasion, and survival [63]. In our study, oxalate increased mitochondrial respiration, ATP production, and mitochondrial activity in 786-O cells but did not alter these endpoints in 769-P cells. Further, 786-O cells had a dose dependent increase in glycolysis; whereas, only the highest CaOx concentration stimulated glycolysis in 769-P cells. Collectively, the rise in respiration and glycolysis in 786-O cells suggest that oxalate promotes metabolic flexibility, rapid cell growth, and survival, potentially deviating from the classical Warburg effect often observed in ccRCC [64]. However, confirming this will require future ^13^C‑glucose tracing studies.

Based on these findings, we postulated that ROS may be involved in these phenomena. A delicate balance of intracellular ROS is essential, as high ROS generation can lead to DNA damage and cell death. In contrast, low to moderate levels can promote cell proliferation, signaling, and migration [65, 66]. Because ROS can promote DNA damage and tumorigenic properties in cells, we evaluated ROS and DNA damage. In 786-O cells, both forms of oxalate lowered total ROS levels and NaOx reduced DNA damage. Conversely, CaOx crystals increased DNA damage in 786-O cells, which could reflect physical crystal and cell interactions or the release of damage-associated molecular patterns. The reduced DNA damage in NaOx treated cells could be due to the activation of DNA repair pathways, as it has been reported that renal cancer cells can repair DNA damage through DNA damage response pathways [67]. In contrast, oxalate increased ROS levels and DNA damage in 769-P cells.

The differences in ROS production and proliferation between the two cell lines likely reflect their distinct metabolic programs. Prior work shows that 786‑O cells can proliferate despite low ROS levels, which may be due to strong antioxidant buffering and redox-adaptive metabolism [68, 69]. This may explain why low doses of CaOx or NaOx increased growth in 786-O cells without elevating ROS. In contrast, 769‑P cells show modest ROS increases, which is consistent with reports that some ccRCC subtypes rely on ROS signaling to support growth [70]. Indeed, these cell line specific patterns help reconcile the differential responses observed in our study.

To determine if these findings were subtype specific, we used Caki-1 cells, another ccB cell line with distinct phenotypic and transcriptional differences from 786-O cells [71, 72]. Caki‑1 cells are derived from a metastatic lesion, whereas 786‑O cells originate from a primary tumor, and these distinct origins may influence their adaptive and stress‑response, oxygen-sensing, nutrient utilization, and redox [73]. Nevertheless, Caki-1 remains a widely used comparator in ccRCC studies. Many of the observations observed in 786-O cells also occurred in Caki-1 cells following oxalate exposure, including increased metabolism and ATP levels and reduced ROS levels. These shared responses reflect RCC’s metabolic plasticity and the ability of various ccRCC subtypes to maintain redox balance under stress [73]. Because this pilot study was not powered for formal subtype interaction testing, these patterns should be interpreted as initial evidence and will require validation in larger, controlled studies.

A number of cellular pathways are involved in renal cancer, including mTOR, which regulates cell proliferation, mitochondrial function, and metabolism [21, 43]. Additionally, oxalate has been reported to activate mTOR signaling in a human renal epithelial cell line [48]. Based on our findings and these previous reports, we investigated whether oxalate influences mTOR signaling in ccB cell lines. Both 786-O and Caki-1 cell lines express basal mTOR protein expression [74]. While oxalate did not directly induce S6K phosphorylation, rapamycin (mTOR inhibitor) restored metabolism and ATP levels to control cells in both ccB subtypes. This suggests that CaOx may alter other pathways downstream or parallel of mTOR signaling. Indeed, CaOx affects autophagy and mitochondrial activity in renal tubular cells, potentially through TFEB localization and ULK1 phosphorylation [48]. Alternatively, CaOx may induce a secondary pathway such as AMPK or HIF1-alpha that is bypassed by mTOR inhibition [75]. Taken together, more studies are needed to define the specific mechanisms involved.

The loss of VHL in ccRCC, together with our findings, suggest that VHL-HIF-mTOR crosstalk may occur. This signaling axis integrates oxygen sensing and oxidative stress with metabolism and cell cycle control [76]. Moderate increases in ROS can stabilize HIF and support mTOR signaling, promoting glycolysis, mitochondrial respiration, and S-phase entry; whereas, higher ROS levels can suppress mTOR and promote apoptotic signaling. The dose-dependent effects of oxalate in our study, align with the VHL-HIF-mTOR axis. While this axis provides useful context, its detailed investigation was beyond the scope of this pilot study.

## Conclusions

Our findings suggest that oxalate may contribute to the association between ccRCC and KS disease, providing new insight into potential metabolic links connecting these conditions. We show that ccRCC cells respond differently to oxalate based on its form, concentration, and tumor subtype. These subtype patterns highlight oxalate-mediated metabolic reprogramming. The purpose of this pilot study was to determine whether oxalate impacts ccRCC metabolic responses using human cell lines with distinct metastatic responses, and our results provide initial evidence that oxalate can modulate RCC energy metabolism and redox signaling.

We acknowledge some study limitations. We did not evaluate mitochondrial protein levels to validate transcript changes, which could identify potential post-translational modifications [77–79]. Our primary goal was to assess immediate mitochondrial mRNA responses, rather than to define the entire biogenesis pathway or post‑translational protein effects. Additionally, the basal activity in these cell lines slightly differ and could impact interpretation. This study was not powered for formal subtype specific effects nor did we perform time course experiments. We also did not examine the antioxidant response or upstream or additional downstream PI3/AKT/mTOR signaling. Since dissecting this pathway can be quite complex [80, 81], our objective was to assess whether mTOR signaling is responsive to oxalate.

Despite these limitations, our preliminary findings highlight an intriguing connection between oxalate and ccRCC metabolism. Further research is needed to elucidate additional underlying mechanisms and their potential clinical implications. Future studies will examine specific cellular pathways involved and whether oxalate can impact ccRCC *in vivo*. Additional studies focused on larger kidney stone and ccRCC cohort analyses will also provide translational relevance. Understanding these interconnected processes in more depth, could identify novel therapeutic targets to enhance current treatments and improve outcomes for patients with ccRCC.

## Supplementary Information

Supplementary Material 1.

Supplementary Material 2.

## Data Availability

The datasets used and/or analyzed during the current study are available from the corresponding author on reasonable request.

## Abbreviations

ATP: Adenosine triphosphate
BAD: BCL2 associated agonist of cell death
BAX: BCL2-like protein 4
CaOx: Calcium oxalate
ccRCC: Clear cell renal cell carcinoma
ccA: Clear cell type A
ccB: Clear cell type B
COX-1: Cyclooxygenase-1
COX-4: Cyclooxygenase-4
ECAR: Extracellular acidification rate
FASN: Fatty acid synthase
FH: Fumarate Hydratase
G1: Gap 1 phase of the cell cycle
G2: Gap 2 phase of the cell cycle
HIF: Hypoxia-inducible factor
IDH: Isocitrate dehydrogenase
KS: Kidney stones
M: M phase of the cell cycle or mitosis
MMP: Mitochondrial membrane potential
NaOx: Sodium oxalate
OCR: Oxygen consumption rate
PGC-1α: Peroxisome proliferator-activated receptor gamma coactivator 1 alpha
PI: Propidium iodide
RADD: Repair Assisted Damage Detection
RCC: Renal cell carcinoma
ROS: Reactive oxygen species
S: Synthesis phase of the cell cycle
SDHA: Succinate dehydrogenase
TCA: Tricarboxylic acid cycle
TFAM: Mitochondrial Transcription Factor A
