# Supplementary material for: Oxalate induces proliferation and mitochondrial metabolism in select clear cell renal cell carcinoma cell lines
Source: BMC Cancer. 2026 Mar 17;26:520. doi: 10.1186/s12885-026-15847-0 (PMC13107611; doi:10.1186/s12885-026-15847-0)
Supplement: Supplementary file 1 — Supplementary Material 1. [file 12885_2026_15847_MOESM1_ESM.pptx]

## Slide 1
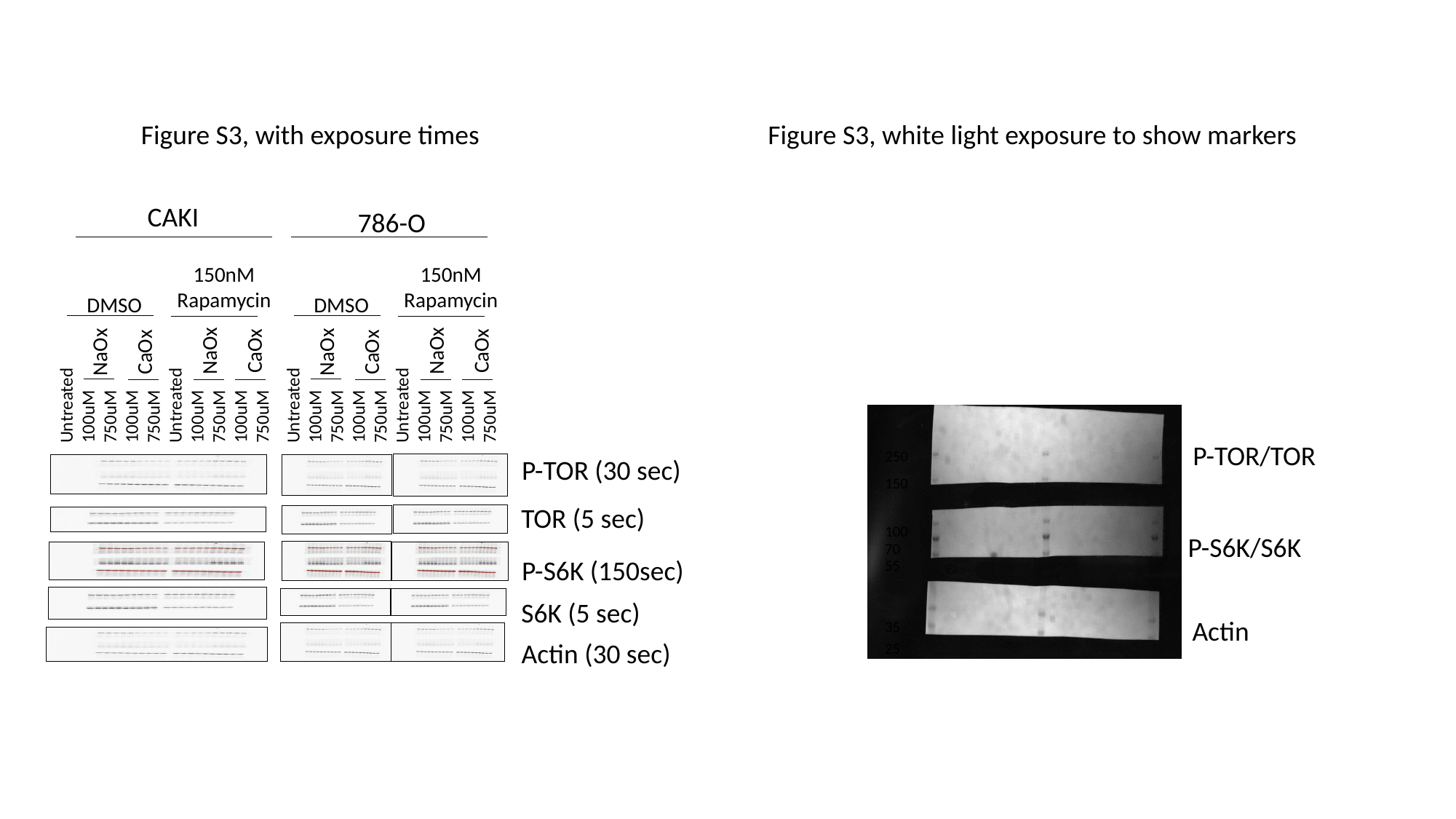

Figure S3, with exposure times
Figure S3, white light exposure to show markers
CAKI
786-O
150nM
Rapamycin
150nM
Rapamycin
Untreated
100uM
750uM
100uM
750uM
Untreated
100uM
750uM
100uM
750uM
Untreated
100uM
750uM
100uM
750uM
Untreated
100uM
750uM
100uM
750uM
DMSO
DMSO
CaOx
NaOx
CaOx
NaOx
CaOx
NaOx
CaOx
NaOx
P-TOR/TOR
250
P-TOR (30 sec)
150
TOR (5 sec)
100
P-S6K/S6K
70
P-S6K (150sec)
55
S6K (5 sec)
Actin
35
Actin (30 sec)
25

## Slide 2
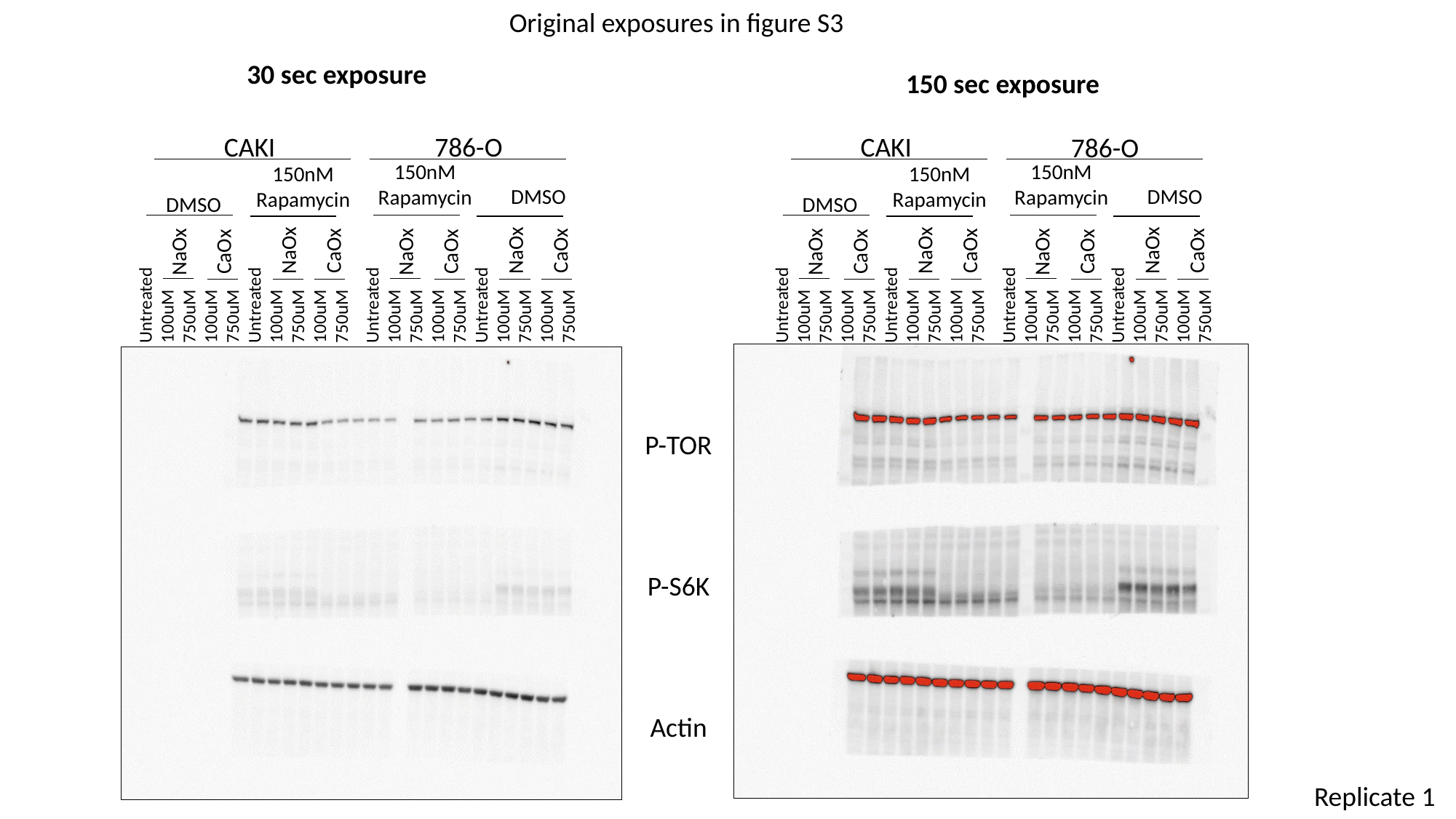

Original exposures in figure S3
30 sec exposure
150 sec exposure
786-O
CAKI
CAKI
786-O
150nM
Rapamycin
150nM
Rapamycin
150nM
Rapamycin
150nM
Rapamycin
Untreated
100uM
750uM
100uM
750uM
Untreated
100uM
750uM
100uM
750uM
Untreated
100uM
750uM
100uM
750uM
Untreated
100uM
750uM
100uM
750uM
Untreated
100uM
750uM
100uM
750uM
Untreated
100uM
750uM
100uM
750uM
Untreated
100uM
750uM
100uM
750uM
Untreated
100uM
750uM
100uM
750uM
DMSO
DMSO
DMSO
DMSO
CaOx
CaOx
NaOx
CaOx
NaOx
CaOx
NaOx
NaOx
CaOx
CaOx
NaOx
CaOx
NaOx
CaOx
NaOx
NaOx
P-TOR
P-S6K
Actin
Replicate 1

## Slide 3
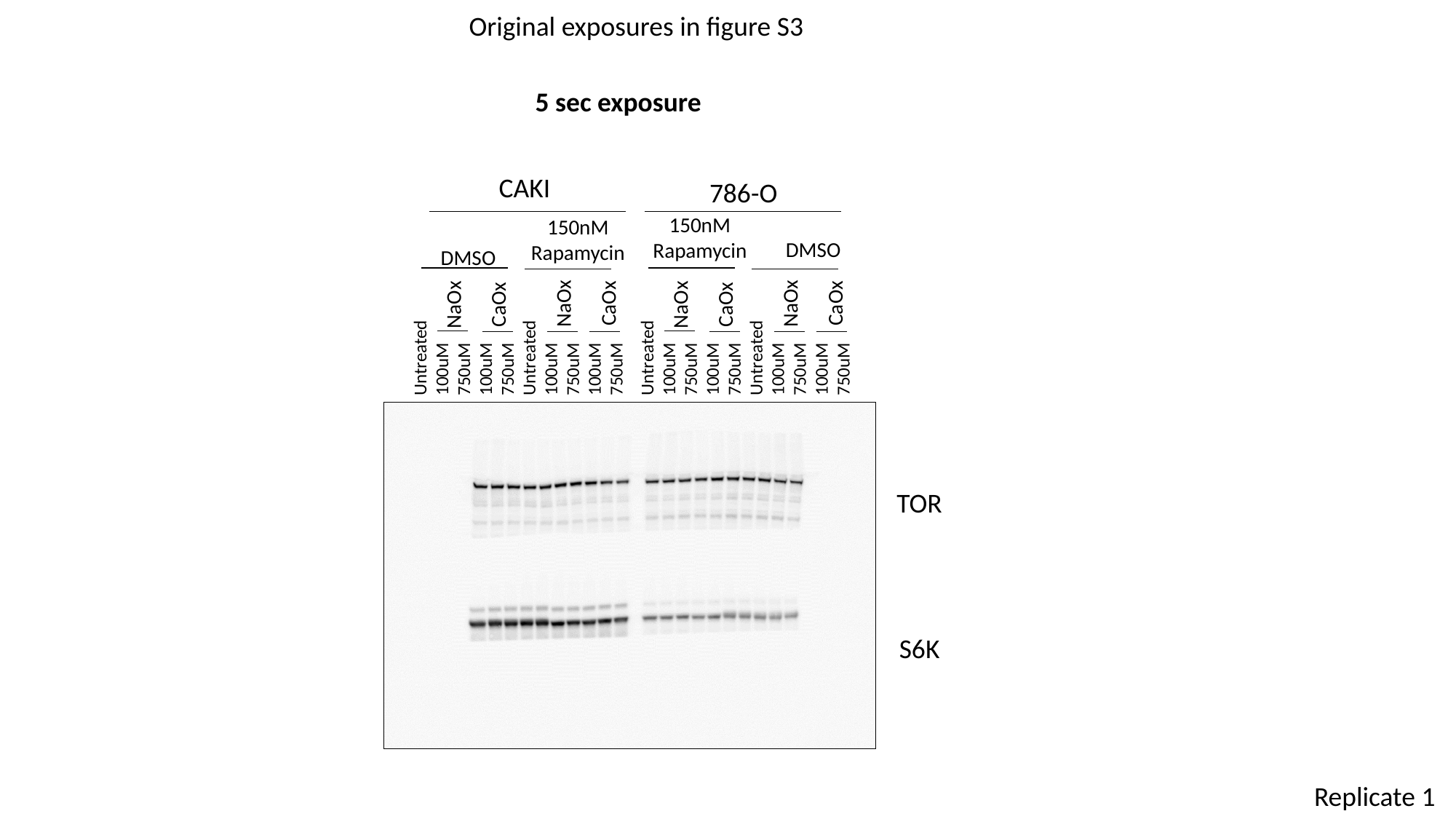

Original exposures in figure S3
5 sec exposure
CAKI
786-O
150nM
Rapamycin
150nM
Rapamycin
Untreated
100uM
750uM
100uM
750uM
Untreated
100uM
750uM
100uM
750uM
Untreated
100uM
750uM
100uM
750uM
Untreated
100uM
750uM
100uM
750uM
DMSO
DMSO
CaOx
NaOx
CaOx
NaOx
CaOx
NaOx
CaOx
NaOx
TOR
S6K
Replicate 1

## Slide 4
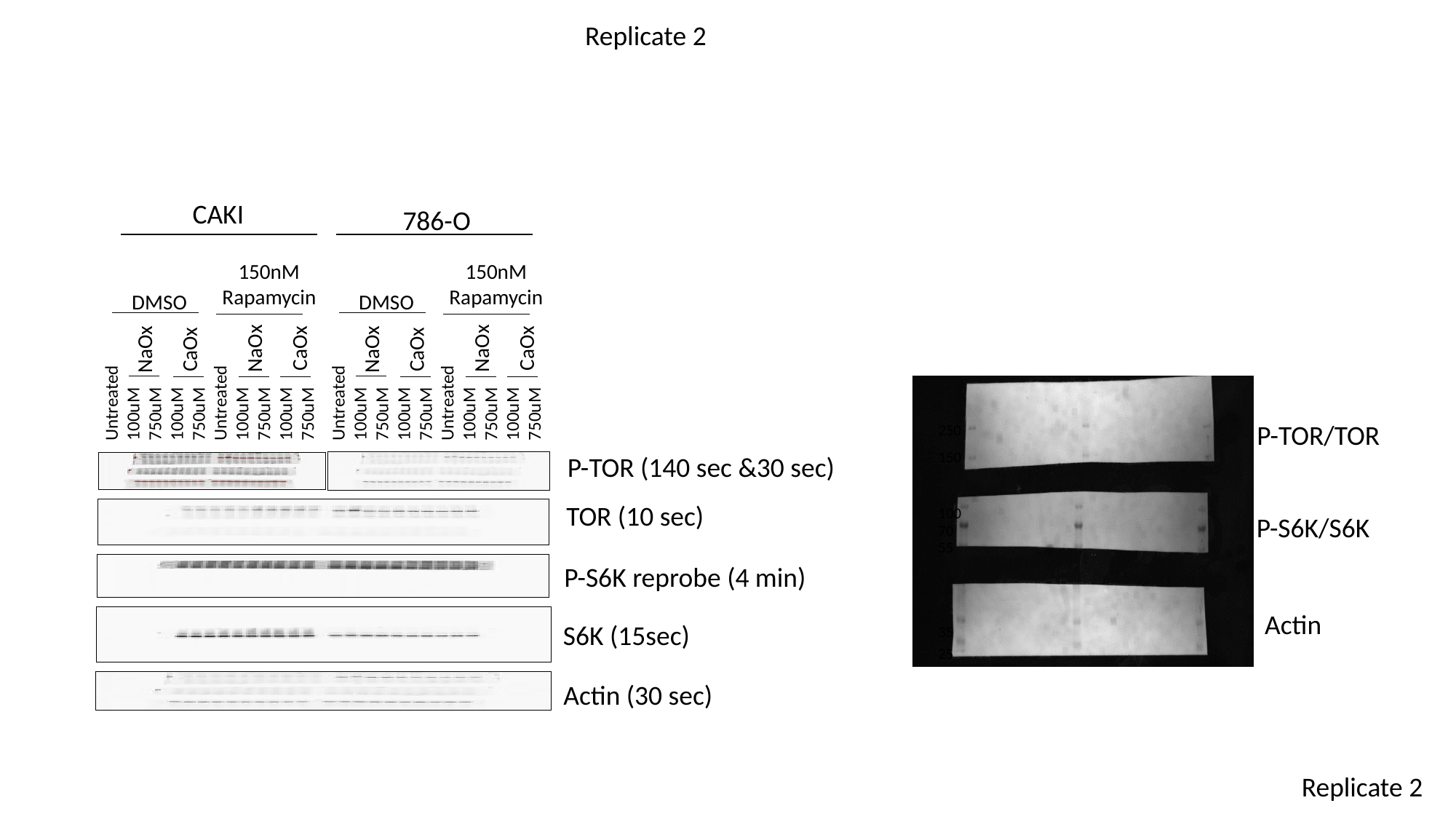

Replicate 2
CAKI
786-O
150nM
Rapamycin
150nM
Rapamycin
Untreated
100uM
750uM
100uM
750uM
Untreated
100uM
750uM
100uM
750uM
Untreated
100uM
750uM
100uM
750uM
Untreated
100uM
750uM
100uM
750uM
DMSO
DMSO
CaOx
NaOx
CaOx
NaOx
CaOx
NaOx
CaOx
NaOx
P-TOR/TOR
250
150
P-TOR (140 sec &30 sec)
TOR (10 sec)
100
P-S6K/S6K
70
55
P-S6K reprobe (4 min)
Actin
S6K (15sec)
35
25
Actin (30 sec)
Replicate 2

## Slide 5
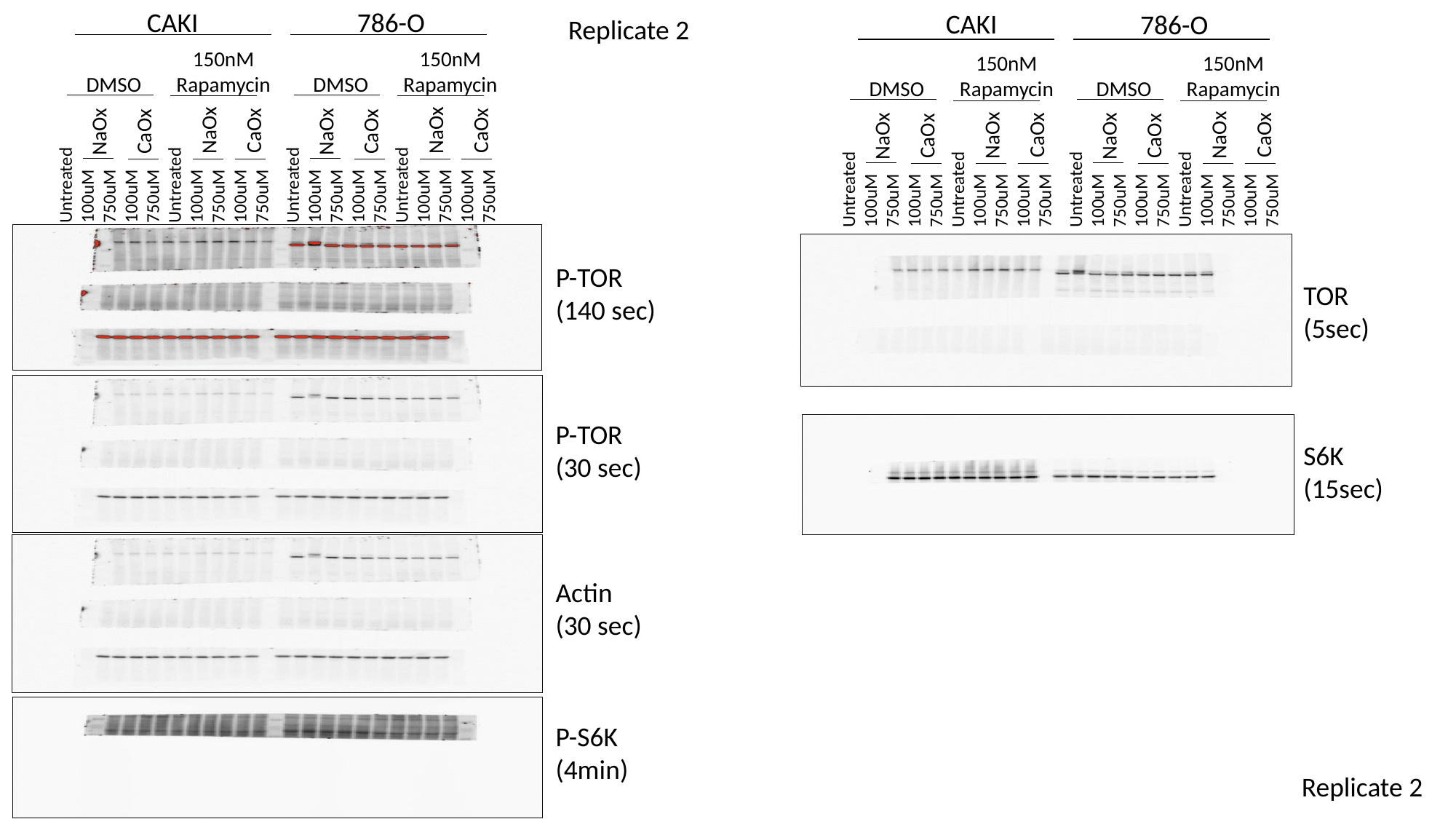

CAKI
786-O
CAKI
786-O
Replicate 2
150nM
Rapamycin
150nM
Rapamycin
150nM
Rapamycin
150nM
Rapamycin
Untreated
100uM
750uM
100uM
750uM
Untreated
100uM
750uM
100uM
750uM
Untreated
100uM
750uM
100uM
750uM
Untreated
100uM
750uM
100uM
750uM
Untreated
100uM
750uM
100uM
750uM
Untreated
100uM
750uM
100uM
750uM
Untreated
100uM
750uM
100uM
750uM
Untreated
100uM
750uM
100uM
750uM
DMSO
DMSO
DMSO
DMSO
CaOx
NaOx
CaOx
NaOx
CaOx
NaOx
CaOx
NaOx
CaOx
NaOx
CaOx
NaOx
CaOx
NaOx
CaOx
NaOx
P-TOR
(140 sec)
TOR
(5sec)
P-TOR
(30 sec)
S6K
(15sec)
Actin
(30 sec)
P-S6K
(4min)
Replicate 2

## Slide 6
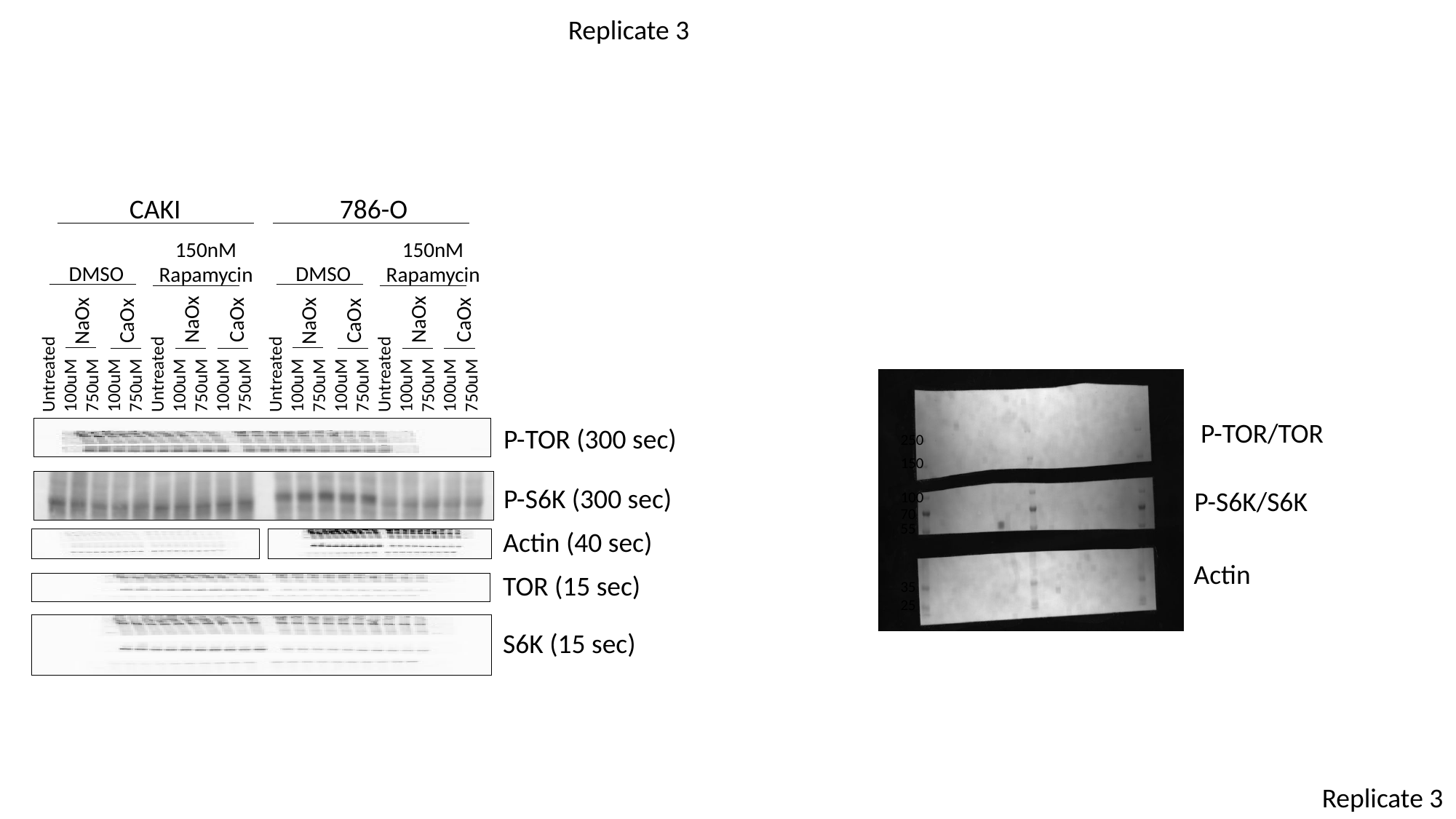

Replicate 3
CAKI
786-O
150nM
Rapamycin
150nM
Rapamycin
Untreated
100uM
750uM
100uM
750uM
Untreated
100uM
750uM
100uM
750uM
Untreated
100uM
750uM
100uM
750uM
Untreated
100uM
750uM
100uM
750uM
DMSO
DMSO
CaOx
NaOx
CaOx
NaOx
CaOx
NaOx
CaOx
NaOx
P-TOR/TOR
P-TOR (300 sec)
250
150
P-S6K (300 sec)
P-S6K/S6K
100
70
55
Actin (40 sec)
Actin
TOR (15 sec)
35
25
S6K (15 sec)
Replicate 3

## Slide 7
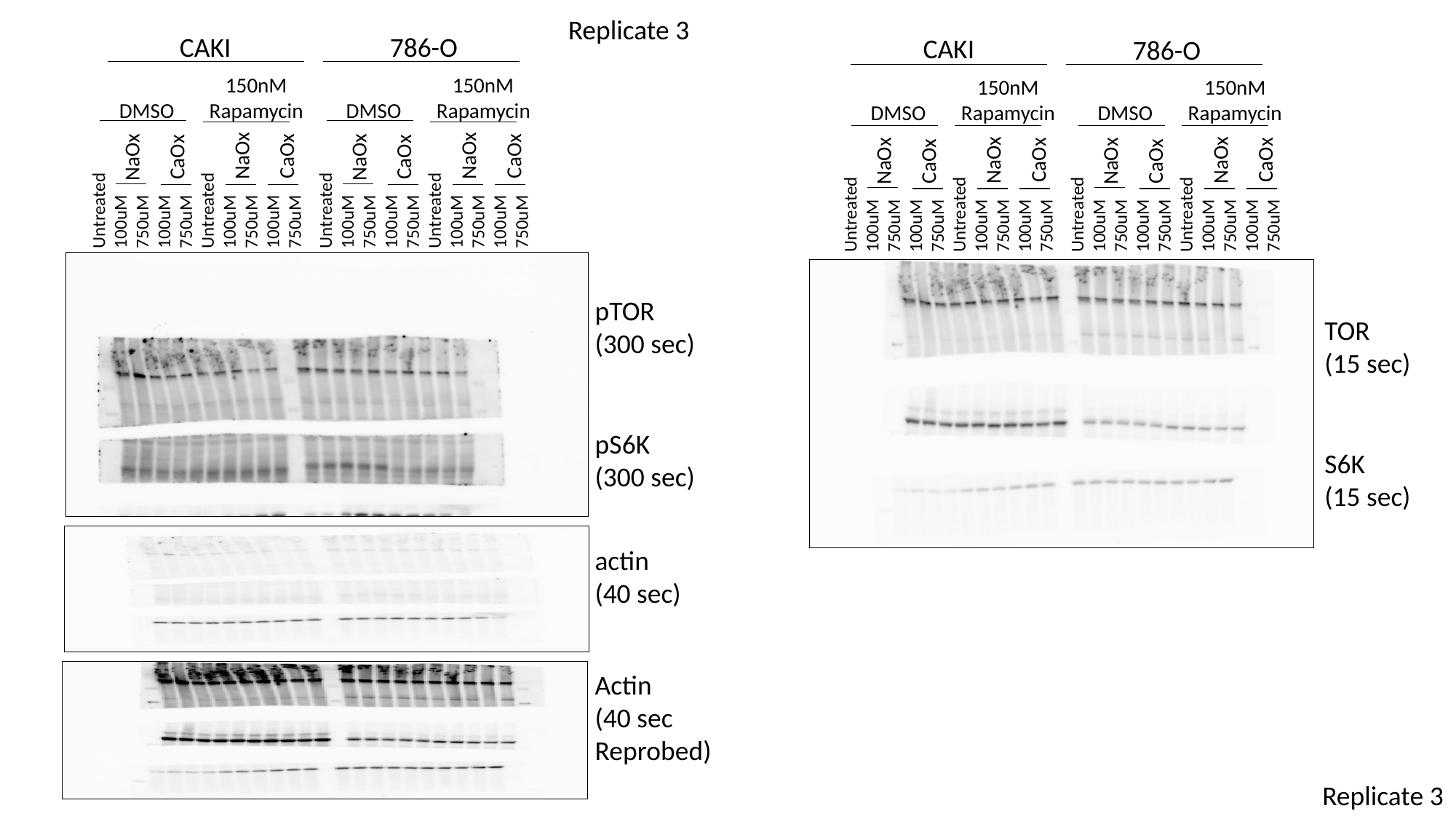

Replicate 3
CAKI
786-O
CAKI
786-O
150nM
Rapamycin
150nM
Rapamycin
150nM
Rapamycin
150nM
Rapamycin
Untreated
100uM
750uM
100uM
750uM
Untreated
100uM
750uM
100uM
750uM
Untreated
100uM
750uM
100uM
750uM
Untreated
100uM
750uM
100uM
750uM
Untreated
100uM
750uM
100uM
750uM
Untreated
100uM
750uM
100uM
750uM
Untreated
100uM
750uM
100uM
750uM
Untreated
100uM
750uM
100uM
750uM
DMSO
DMSO
DMSO
DMSO
CaOx
NaOx
CaOx
NaOx
CaOx
NaOx
CaOx
NaOx
CaOx
NaOx
CaOx
NaOx
CaOx
NaOx
CaOx
NaOx
pTOR
(300 sec)
TOR
(15 sec)
pS6K
(300 sec)
S6K
(15 sec)
actin
(40 sec)
Actin
(40 sec
Reprobed)
Replicate 3
