# Supplementary material for: Oxalate induces proliferation and mitochondrial metabolism in select clear cell renal cell carcinoma cell lines
Source: BMC Cancer. 2026 Mar 17;26:520. doi: 10.1186/s12885-026-15847-0 (PMC13107611; doi:10.1186/s12885-026-15847-0)
Supplement: Supplementary file 2 — Supplementary Material 2. [file 12885_2026_15847_MOESM2_ESM.pptx]

## Slide 1
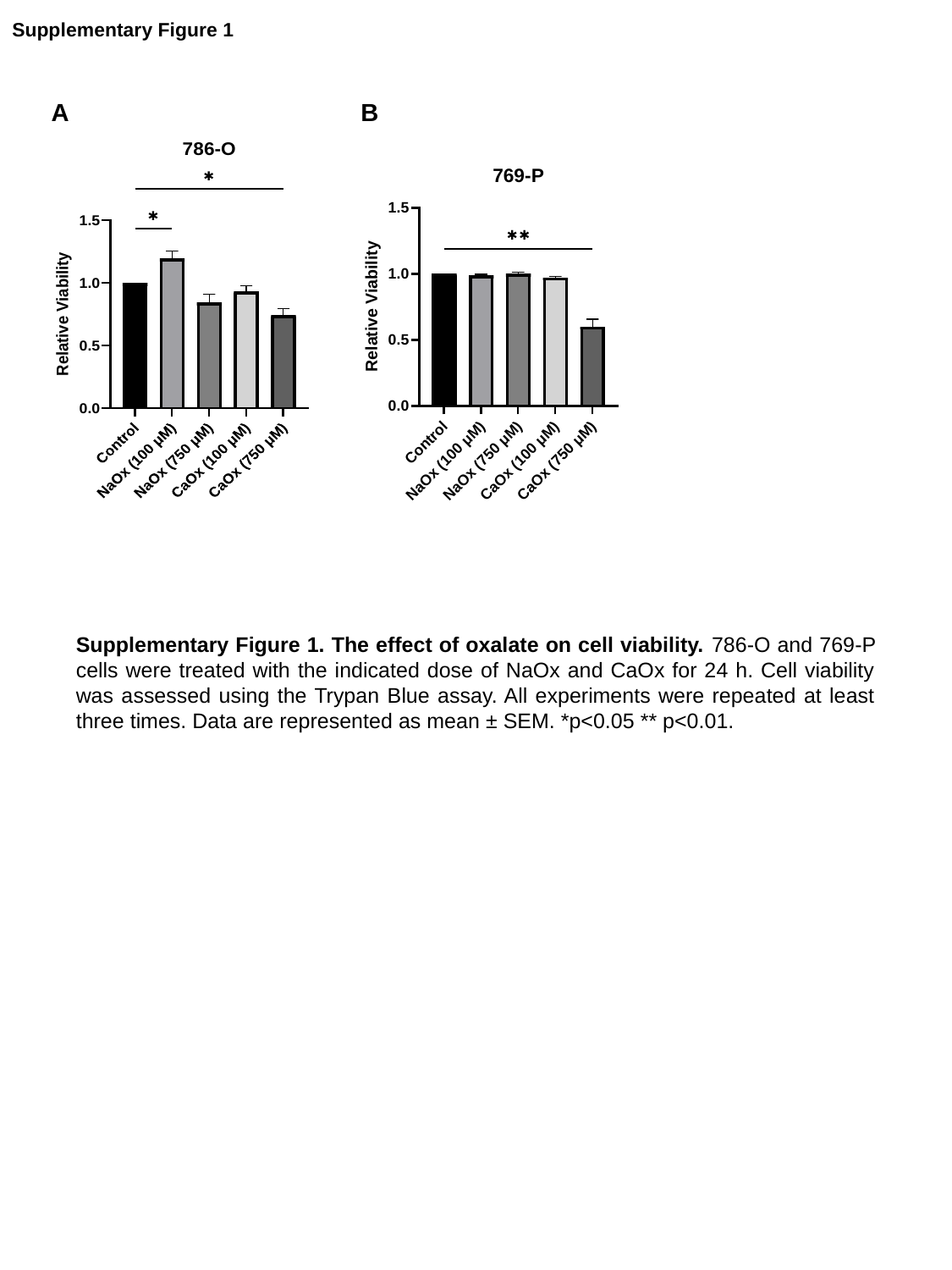

Supplementary Figure 1
A
B
Supplementary Figure 1. The effect of oxalate on cell viability. 786-O and 769-P cells were treated with the indicated dose of NaOx and CaOx for 24 h. Cell viability was assessed using the Trypan Blue assay. All experiments were repeated at least three times. Data are represented as mean ± SEM. *p<0.05 ** p<0.01.

## Slide 2
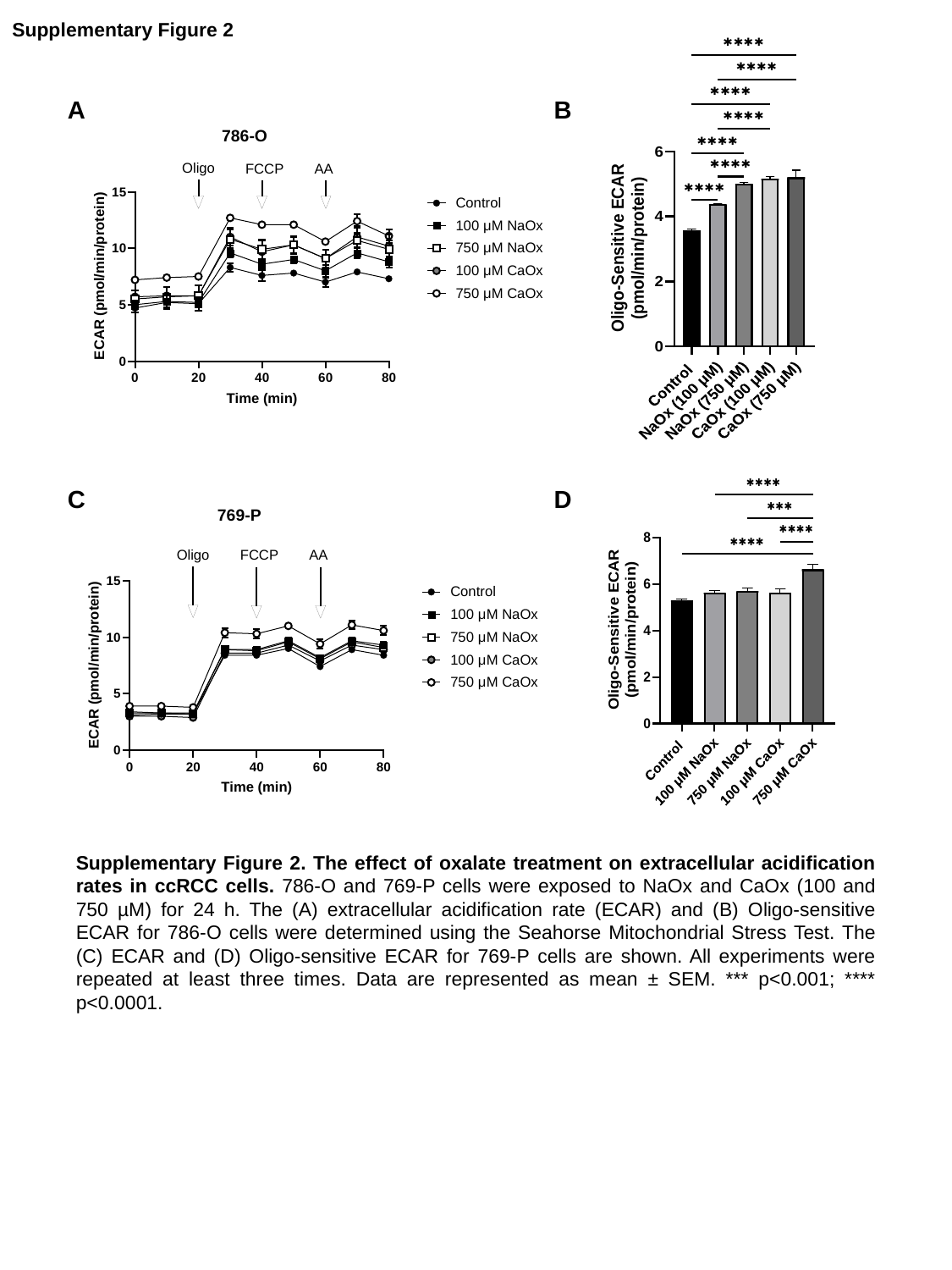

Supplementary Figure 2
A
B
C
D
Supplementary Figure 2. The effect of oxalate treatment on extracellular acidification rates in ccRCC cells. 786-O and 769-P cells were exposed to NaOx and CaOx (100 and 750 µM) for 24 h. The (A) extracellular acidification rate (ECAR) and (B) Oligo-sensitive ECAR for 786-O cells were determined using the Seahorse Mitochondrial Stress Test. The (C) ECAR and (D) Oligo-sensitive ECAR for 769-P cells are shown. All experiments were repeated at least three times. Data are represented as mean ± SEM. *** p<0.001; **** p<0.0001.

## Slide 3
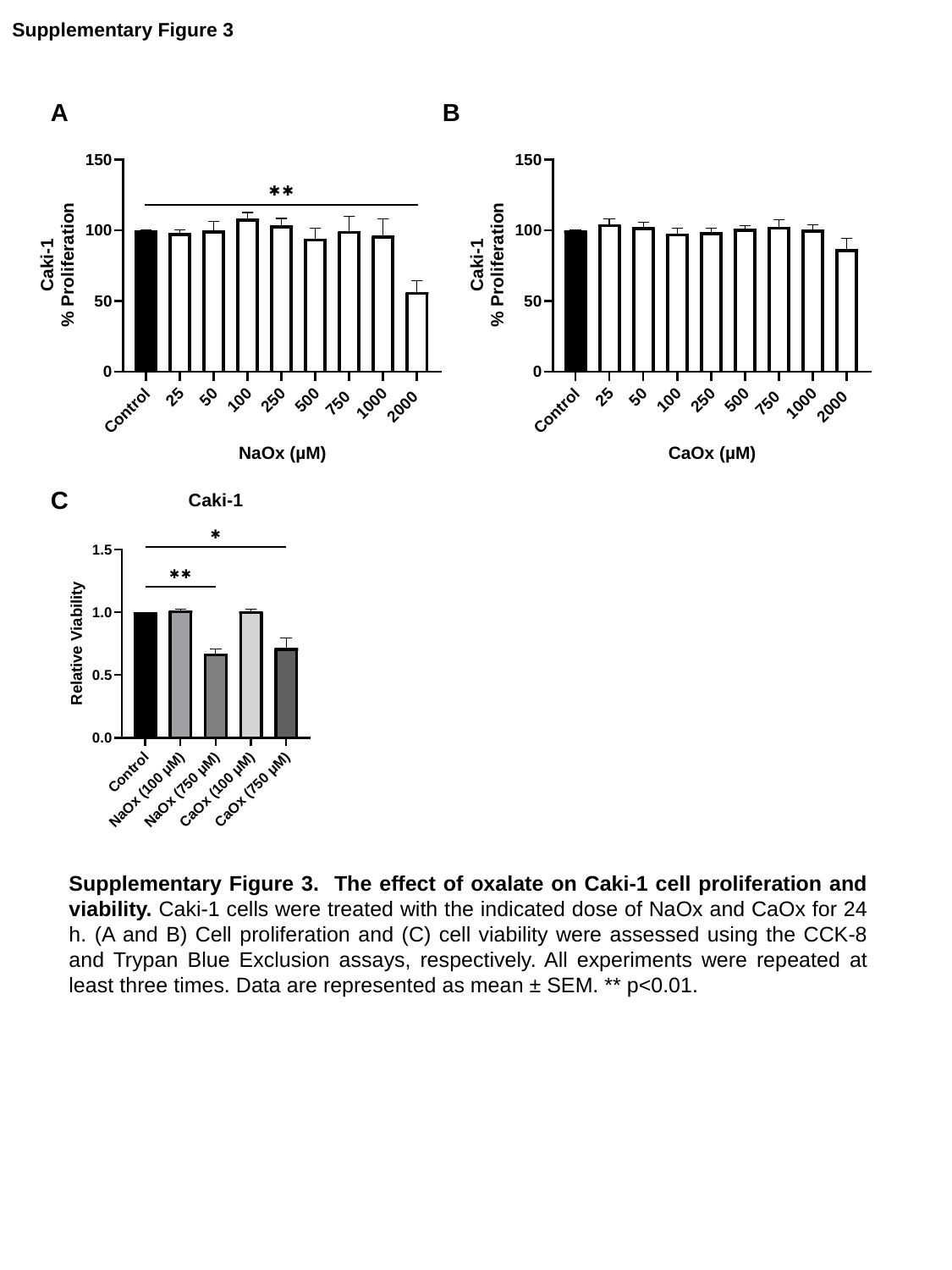

Supplementary Figure 3
A
B
C
Supplementary Figure 3. The effect of oxalate on Caki-1 cell proliferation and viability. Caki-1 cells were treated with the indicated dose of NaOx and CaOx for 24 h. (A and B) Cell proliferation and (C) cell viability were assessed using the CCK-8 and Trypan Blue Exclusion assays, respectively. All experiments were repeated at least three times. Data are represented as mean ± SEM. ** p<0.01.

## Slide 4
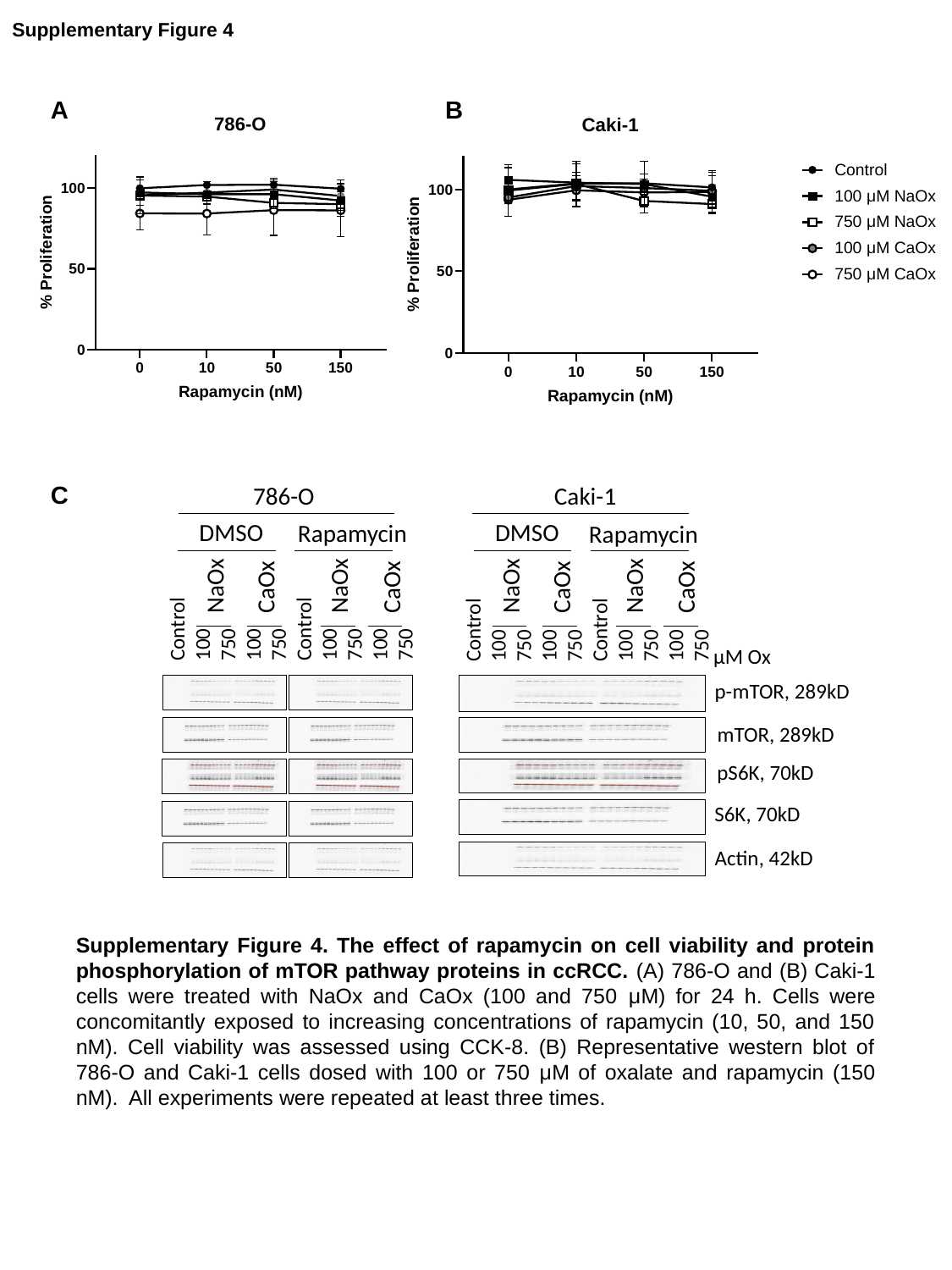

Supplementary Figure 4
B
A
C
786-O
Caki-1
Control
100
750
100
750
Control
100
750
100
750
Control
100
750
100
750
Control
100
750
100
750
DMSO
DMSO
Rapamycin
Rapamycin
NaOx
NaOx
NaOx
NaOx
CaOx
CaOx
CaOx
CaOx
μM Ox
p-mTOR, 289kD
mTOR, 289kD
pS6K, 70kD
S6K, 70kD
Actin, 42kD
Supplementary Figure 4. The effect of rapamycin on cell viability and protein phosphorylation of mTOR pathway proteins in ccRCC. (A) 786-O and (B) Caki-1 cells were treated with NaOx and CaOx (100 and 750 μM) for 24 h. Cells were concomitantly exposed to increasing concentrations of rapamycin (10, 50, and 150 nM). Cell viability was assessed using CCK-8. (B) Representative western blot of 786-O and Caki-1 cells dosed with 100 or 750 μM of oxalate and rapamycin (150 nM). All experiments were repeated at least three times.

## Slide 5
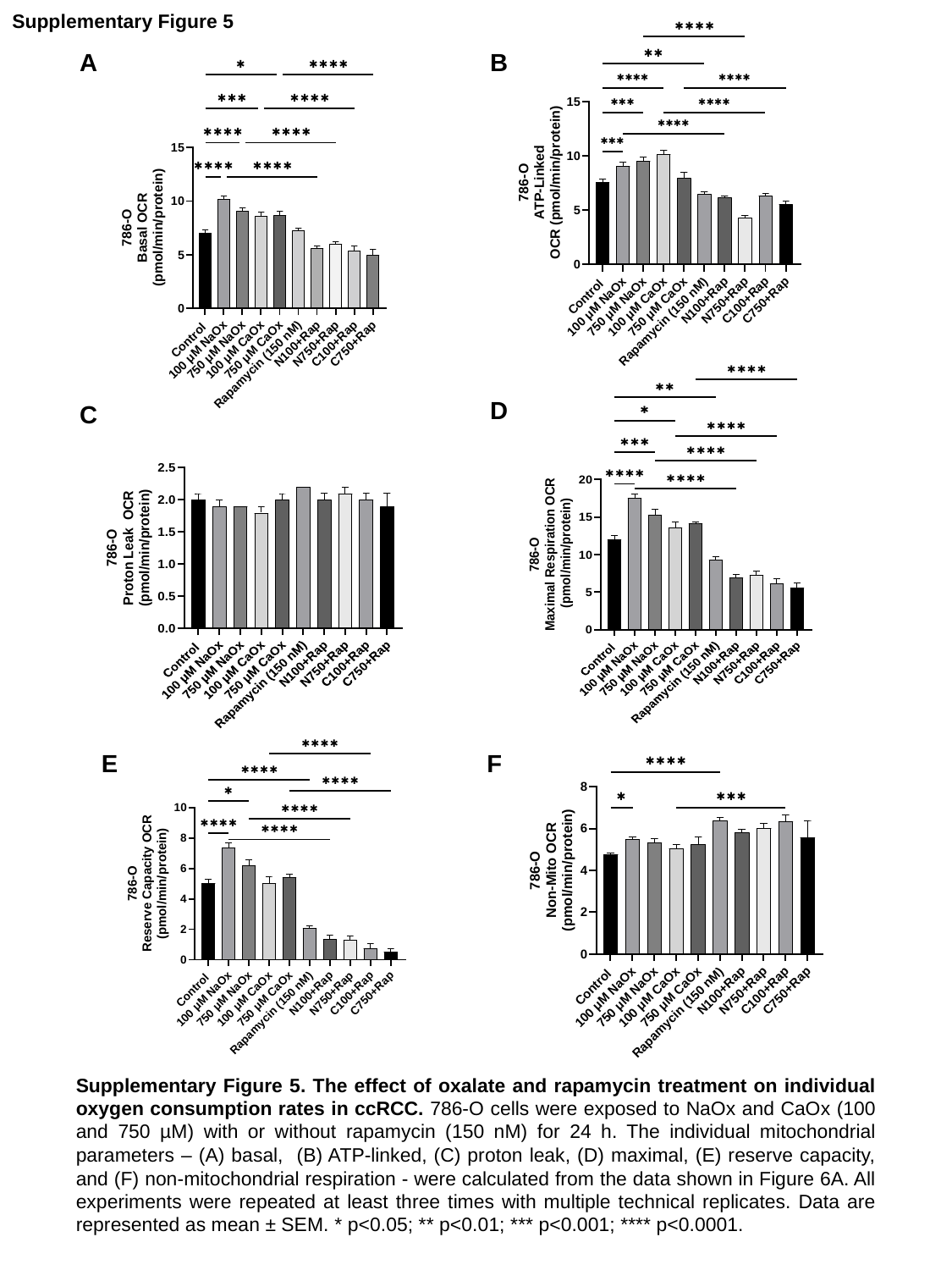

Supplementary Figure 5
A
B
D
C
E
F
Supplementary Figure 5. The effect of oxalate and rapamycin treatment on individual oxygen consumption rates in ccRCC. 786-O cells were exposed to NaOx and CaOx (100 and 750 µM) with or without rapamycin (150 nM) for 24 h. The individual mitochondrial parameters – (A) basal, (B) ATP-linked, (C) proton leak, (D) maximal, (E) reserve capacity, and (F) non-mitochondrial respiration - were calculated from the data shown in Figure 6A. All experiments were repeated at least three times with multiple technical replicates. Data are represented as mean ± SEM. * p<0.05; ** p<0.01; *** p<0.001; **** p<0.0001.

## Slide 6
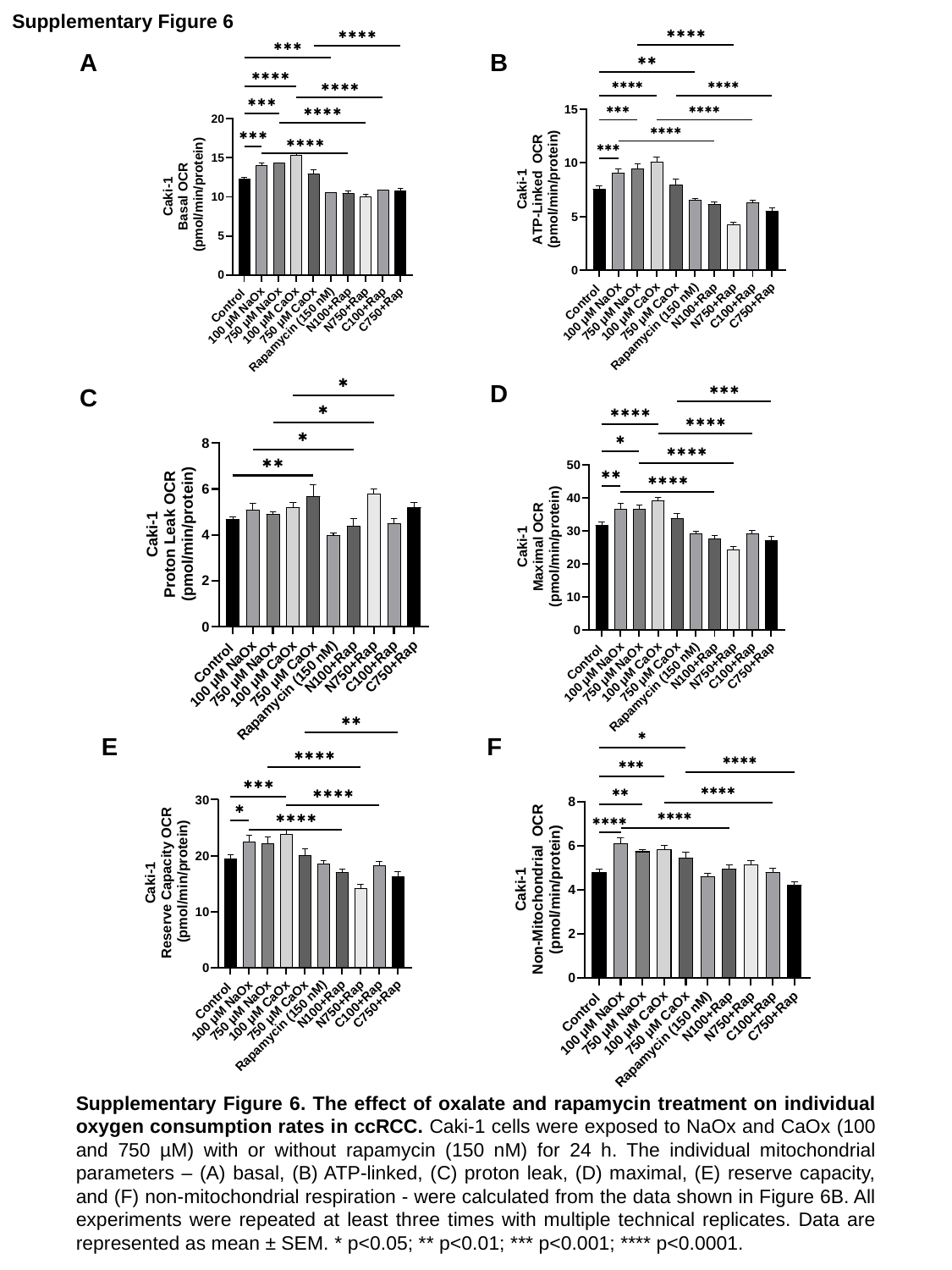

Supplementary Figure 6
A
B
D
C
E
F
Supplementary Figure 6. The effect of oxalate and rapamycin treatment on individual oxygen consumption rates in ccRCC. Caki-1 cells were exposed to NaOx and CaOx (100 and 750 µM) with or without rapamycin (150 nM) for 24 h. The individual mitochondrial parameters – (A) basal, (B) ATP-linked, (C) proton leak, (D) maximal, (E) reserve capacity, and (F) non-mitochondrial respiration - were calculated from the data shown in Figure 6B. All experiments were repeated at least three times with multiple technical replicates. Data are represented as mean ± SEM. * p<0.05; ** p<0.01; *** p<0.001; **** p<0.0001.

## Slide 7
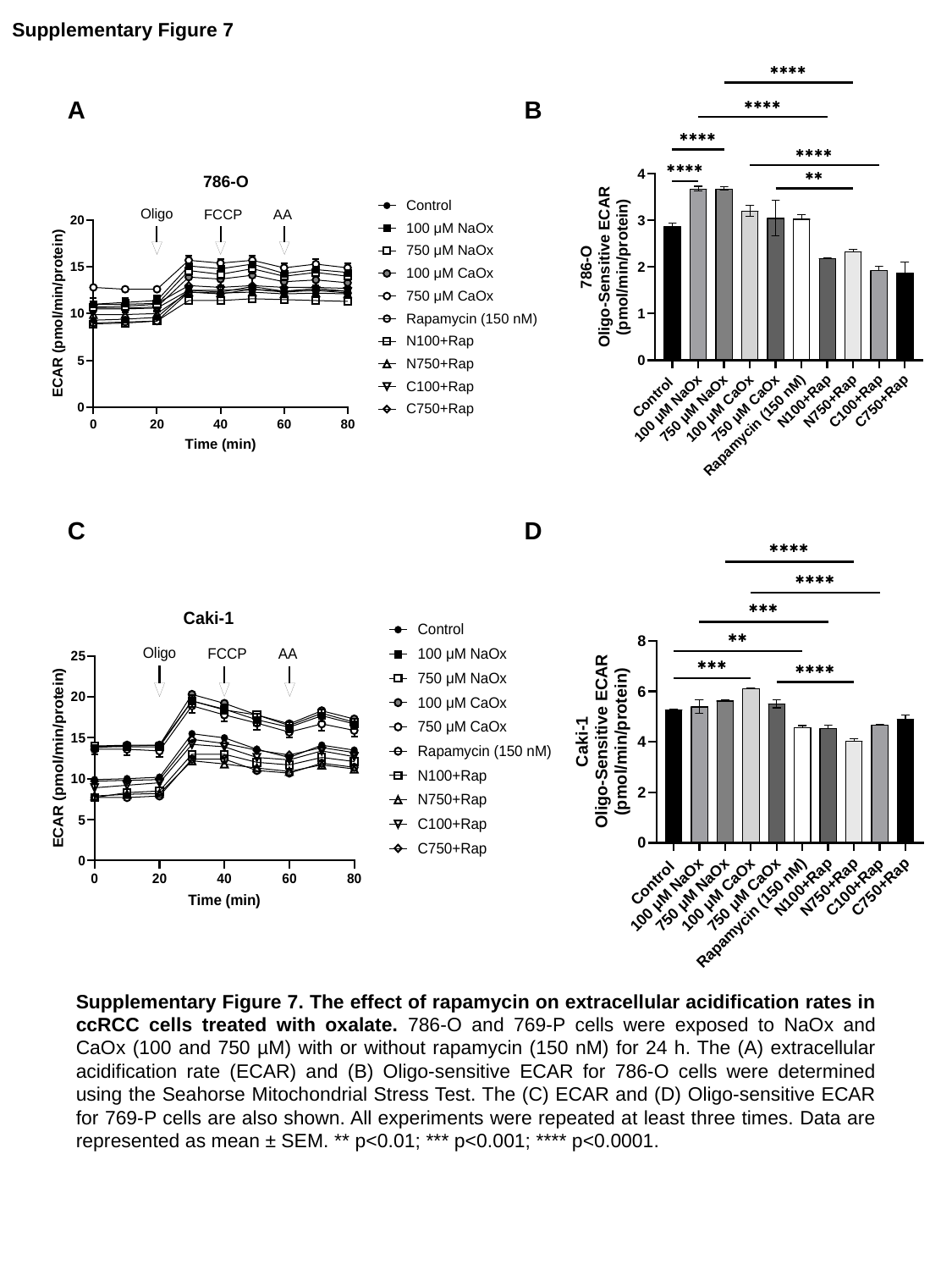

Supplementary Figure 7
A
B
C
D
Supplementary Figure 7. The effect of rapamycin on extracellular acidification rates in ccRCC cells treated with oxalate. 786-O and 769-P cells were exposed to NaOx and CaOx (100 and 750 µM) with or without rapamycin (150 nM) for 24 h. The (A) extracellular acidification rate (ECAR) and (B) Oligo-sensitive ECAR for 786-O cells were determined using the Seahorse Mitochondrial Stress Test. The (C) ECAR and (D) Oligo-sensitive ECAR for 769-P cells are also shown. All experiments were repeated at least three times. Data are represented as mean ± SEM. ** p<0.01; *** p<0.001; **** p<0.0001.
